# Saponins of *Paris polyphylla* for the Improvement of Acne: Anti-Inflammatory, Antibacterial, Antioxidant and Immunomodulatory Effects

**DOI:** 10.3390/molecules29081793

**Published:** 2024-04-15

**Authors:** Luyao Li, Jiachan Zhang, Wenjing Cheng, Feiqian Di, Changtao Wang, Quan An

**Affiliations:** 1 College of Light Industry Science and Engineering, Beijing Technology & Business University, Beijing 100048, China; 2130042093@st.btbu.edu.cn (L.L.); 13581568791@163.com (W.C.); 2130042081@st.btbu.edu.cn (F.D.); wangct@btbu.edu.cn (C.W.); 2 Beijing Key Laboratory of Plant Resource Research and Development, Beijing 100048, China; 3 Institute of Cosmetic Regulatory Science, Beijing 100048, China; 4 Yunnan Baiyao Group Co., Ltd., Kunming 650000, China; anquan@ynbyjk.com

**Keywords:** acne, *P. polyphylla* saponin, anti-inflammatory, antibacterial, immunomodulatory, extraction process

## Abstract

Acne is a chronic inflammatory skin disease with a recurring nature that seriously impacts patients’ quality of life. Currently, antibiotic resistance has made it less effective in treating acne. However, *Paris polyphylla* (*P. polyphylla*) is a valuable medicinal plant with a wide range of chemical components. Of these, *P. polyphylla* saponins modulate the effects in vivo and in vitro through antibacterial, anti-inflammatory, immunomodulatory, and antioxidant effects. Acne is primarily associated with inflammatory reactions, abnormal sebum function, micro-ecological disorders, hair follicle hyperkeratosis, and, in some patients, immune function. Therefore, the role of *P. polyphylla* saponins and their values in treating acne is worthy of investigation. Overall, this review first describes the distribution and characteristics of *P. polyphylla* and the pathogenesis of acne. Then, the potential mechanisms of *P. polyphylla* saponins in treating acne are listed in detail (reduction in the inflammatory response, antibacterial action, modulation of immune response and antioxidant effects, etc.). In addition, a brief description of the chemical composition of *P. polyphylla* saponins and its available extraction methods are described. We hope this review can serve as a quick and detailed reference for future studies on their potential acne treatment.

## 1. Introduction

Acne vulgaris is one of the most common dermatological diseases, affecting about 650 million people worldwide, with more than 85% of cases being in teenagers [1]. In addition, acne brings patients a significant clinical, psychological, and economic burden. As research into acne treatment has intensified, it has been discovered that the use of antibiotic drugs may cause the development of resistance. In contrast, some medicinal plants are considered to have antibacterial, anti-inflammatory, and immune function effects. Therefore, the use of Nat medicines has become an inevitable trend.

Several studies have found that the use of active plant substances can have a beneficial effect on the treatment of acne, such as some flavonoid components, like catechins in green tea [2], licorice [3], and Chalcone A [4], which have anti-inflammatory properties. Besides, humulone and serpentine in hops [5], aloin contained in aloe vera [6], and rhubarb phenols in cassia seed [7] have shown strong antibacterial effects in the treatment of acne. Furthermore, the saponin-like substances of Shigarou have shown remarkable anti-inflammatory [8], antibacterial [9]. and immunity-boosting effects [10] in the treatment of acne.

**Figure 1:**
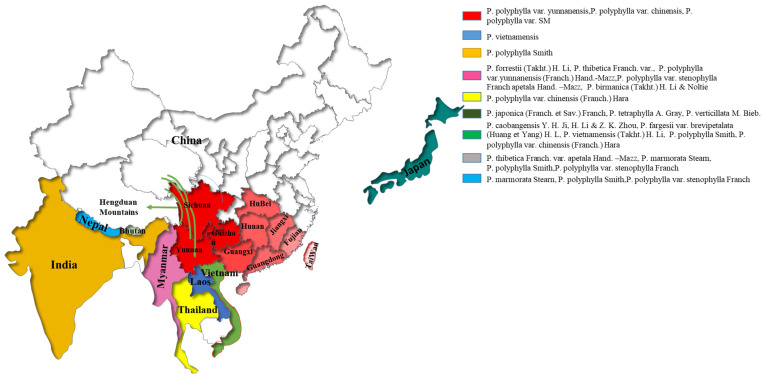
Distribution of Paris genus. Map showing China as the center of Paris species diversity. The green line represents Hengduan Mountain, and the green arrow is a pointing illustration of the Hengduan Mountain.

*Paris polyphylla* belongs to the lily family and Berberidaceae. For over 2000 years, it has been used as an important Nat medicinal plant in China to treat infection, inflammation, and cancer. It was first reported in the “Shennong Materia Medica Classic” [11], and first named Chonglou in the “Dian Nan Ben Cao” [12] and later in Li Shizhen’s “Compendium of Materia Medica” [13]. It was listed in the “Chinensis Pharmacopoeia” for the first time in 1985 [14]. Through years of cultivation and evolution, this herb has been widely distributed in Asian regions such as India [15], China [16], Bhutan, Laos, Myanmar, Nepal [17], Thailand and Vietnam (Figure 1). 

Traditionally, the rhizome of *P. polyphylla* is made into a decoction compatible with other herbal medicines. There are many kinds of prescriptions prepared in this way, such as Chongloujicaishenghua Tang [18], Lianxiuwugong Dilong Tang [19], and Qingwenjiedu soup [20], etc. They have various functions, such as heat-clearing, detoxification, and pain relief, to deal with a sore throat, snake bites, crepitant pain, and convulsions [14]. 

With the development of pharmacological research of *P. polyphylla*, the pharmacological activities, such as anti-tumor [21], antiviral [22], antibacterial [23], antifungal [24], hemostatic [25], and anti-inflammatory [26] effects have been reported and proved. Zhou et al. [21] found that Polyphyllin VII (PP VII) inhibited the differentiation of BMMs into osteoclasts by inhibiting ROS synthesis in vitro. He et al. [27] reported the anti-tumor effect of PP VII by inducing mitochondrial dysfunction and apoptosis and inhibiting PI3K/Akt and NFκB signaling pathways against lung cancer. Wang et al. [22] showed that 95% alcoholic extract of *P. polyphylla* could promote the release of IL-6 and kill the viruses EV71 and CVB3, thus preventing virus replication. Yang et al. [28] found that PPI could exhibit its strong anti-inflammatory ability by regulating the priming pathway of NF-κB and controlling the production of IL-8, IL-6, and TNF-α inflammatory factors. At the same time, it was found that the different types of *P. polyphylla* monomers (PP I, II, III, V, VI, VII and H) showed significant inhibition of the growth of *Propionibacterium acnes* (*P. acnes*), *Staphylococcus epidermidis* (*S. epidermidis*) and *Staphylococcus aureus* (*S. aureus*), and their antibacterial activities were more potent than those of antibiotics [23].

Acne is mainly associated with the proliferation of *P. acnes*, inflammatory response, and abnormal sebaceous gland function [29]. Therefore, early treatment of acne is mainly based on antibiotics and chemicals, which are effective in inhibiting the activity of *P. acnes*, reducing the production of sebum-free fatty acids and extracellular lipase [30], and decreasing the production of inflammatory factors [31]. However, in recent years, studies have found that these chemical acne medications may cause mild to severe side effects, such as skin irritation caused by long-term use of benzoyl peroxide or retinoic acid, including facial skin erythema and peeling [32,33]. Furthermore, treatment may run into issues with biofilm formation or antibiotic-resistant bacteria [34]. Therefore, anti-acne treatment requires the development of safe and clinically effective Nat antibiotics.

Several studies have shown that the secondary metabolites (saponins [8], polysaccharides [35], flavonoids [36], etc.) obtained from *P. polyphylla* have a wide range of biological activities. *P. polyphylla* saponins are the main chemical constituents with anti-inflammatory [8], antibacterial [23], and immunomodulatory [10] effects. The secondary metabolites of steroid saponins can be divided into four groups (isospirostanols, spirostanols, furostanols, and pseudo-spirostanols) which are structurally diverse and have a wide range of active effects. The literature reports that different structures of Paris saponins have different biological activities. Qin et al. [9] suggested that structural variability can significantly affect the bacterial inhibitory capacity of bacteriocin. At the same time, structural differences have different effects on the production of inflammatory mediators [8], regulation of T-cell differentiation [10], keratin regulation [37] and reduction in oxidative stress [38]. As a result, this paper summarizes the role of different structures of *P. polyphylla* saponins in acne treatment and offers additional research ideas for acne treatment.

## 2. Distribution and Characteristics of *P. polyphylla*

*P. polyphylla* plants mainly include about 33 species and 15 varieties [39]. Countries have differences due to temperature, humidity, and species differences. *P. polyphylla* grows widely in temperate and subtropical regions of Europe and Asia in the Northern Hemisphere, mainly in the India, China, Bhutan, Laos, Myanmar, Nepal, Japan, Thailand, Vietnam, and other countries. Among them, *P. polyphylla* in China includes 27 species and 15 varieties [39], of which *P. polyphylla* var. *yunnanensis* and *P. polyphylla* var. *Chinensis* are the most widely distributed and commonly used in China [14]. Figure 1 shows that it is primarily found in China’s Yunnan, Guizhou, and Sichuan provinces. The distribution trend declines from the Hengduan Mountains to the eastern and northeastern Sichuan, Yunnan, and Guizhou provinces [40,41].

**Figure 2:**
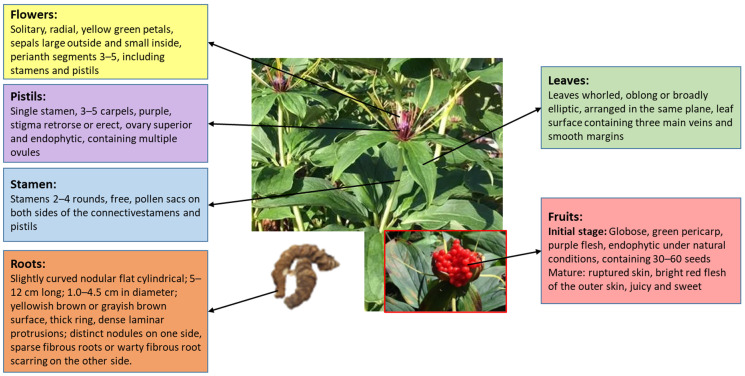
Morphological characteristics of *P. polyphylla*.

*P. polyphylla* is a shade-loving angiosperm with monoecious, unbranched leaves, which are complete with flowers that bloom for up to three months in summer [42] (shown in Figure 2). It grows easily at altitudes of 1300–2500 m or in places with more than 80% canopy closure [43,44], such as bamboo forests, grassy or rocky slopes, streams, coniferous forests, shrublands, and other off-the-beaten-path deep forests or in humus-rich and well-drained soils [44]. In recent years, the survival environment of *P. polyphylla* has been harsh, which makes the germination slow (about 7 months) [45]. Therefore, many researchers have begun to cultivate it in captivity. Different growth years affect the content of effective compounds of *P. polyphylla*. Zhe et al. [46] found that the optimal harvesting time for *P. polyphylla* is 8 years of growth, considering the influence of *P. polyphylla* saponin content and cost factors. Therefore, *P. polyphylla* should be selected and harvested rationally according to the actual needs. 

## 3. Pathogenesis of Acne

**Figure 3:**
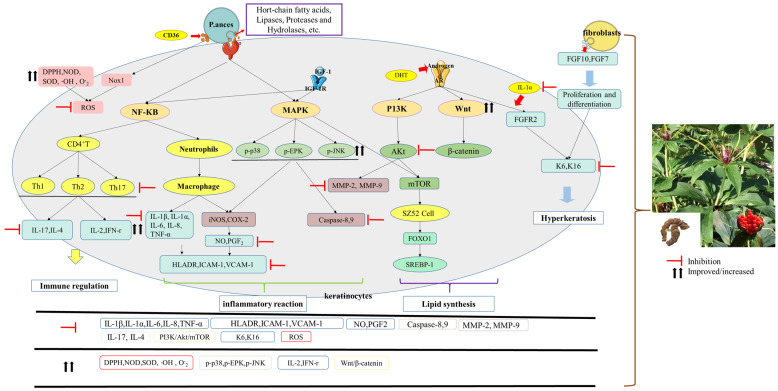
Diagram of the pathogenesis of acne and the mechanism of action of *P. polyphylla*. Inflammatory response, immune regulation, sebum dysfunction, microbial colonization and follicular hyperkeratosis are the driving factors in the development of acne. Peptidoglycan from *P. acnes* stimulates signaling in signaling pathways by binding to TLR2, promoting the differentiation of monocytes into macrophages, and resulting in another large production of inflammatory factors, inflammatory mediators and angiogenic factors. At the same time, by promoting the differentiation of effector T cells into Th1/2/17, it inhibits the production of IL-2 and IFN-γ and promotes the production of the related factors IL-17 and IL-4, thus causing a disruption of the patient’s immune system. Androgens induce lipid synthesis by upregulating PI3K/AKt/mTOR, downregulating the Wnt/β-catenin signalling pathway and thus stimulating SREBP-1 production. As acne causes the production of IL-1α, which promotes the expression of K6/K16, it promotes the proliferation and differentiation of keratin-forming cells and induces the production of follicular hyperkeratosis. *P. polyphylla* can inhibit the production of inflammatory factors and angiogenic factors by suppressing related signaling, as well as modulating the immune system by promoting the production of IL-2 and IFN-γ and inhibiting IL-17 and IL-4. Secondly, *P. polyphylla* can promote Wnt/β-catenin signaling and inhibit PI3K/AKt signaling, a signaling pathway involved in lipid synthesis. Finally, *P. polyphylla* has an inhibitory effect on K6/K16 and regulates hair follicle hyperkeratosis. The red arrows indicate the promoting effect of *P. polyphylla* and the black arrows indicate the inhibiting effect of *P. polyphylla*.

Acne is a chronic inflammatory skin disorder primarily affecting the face, chest, and back. Severe acne dramatically affects the patient’s emotional health and quality of life. In recent years, a great deal of research has been conducted on acne treatment, in which plant extracts have been used as a popular direction in clinical treatment. Among them, *P. polyphylla* has strong anti-inflammatory and antimicrobial properties, and, thus, has potential anti-acne efficacy. In order to better summarize the anti-acne activities and their mechanisms of *P. polyphylla*, an overview of the four main pathogenic mechanisms of acne is presented, as shown in Figure 3.

### 3.1. Microbial Customization

The skin microbiota is indispensable in keratinocyte maturation and host immune regulation. *S. aureus*, *S. epidermidis*, and *P. acnes* (also known as *Cutibacterium acnes*, *C. acnes*) are microorganisms associated with the pathogenesis of acne [47]. Among them, *P. acnes* is considered to be the primary pathogen causing acne [29].

Current studies have confirmed that *P. acnes* metabolizes short-chain fatty acids [48] (acetate, propionate, and butyrate), produces enzymes [49] (lipases, proteases, and hydrolases) and other bioactive molecules, and hydrolyzes triglycerides in sebum to free fatty acids, thus showing pro-inflammatory and keratinizing effects [50]. Furthermore, *P. acnes* can activate NADPH oxidase 1 (Nox1) on the surface of human keratinocytes by recognizing the receptor for thrombin-activated protein (CD36), promoting the release of reactive oxygen species (ROS) and the secretion of inflammatory factors such as interleukin (IL)-8 [28].

Several studies have found that *P. polyphylla* is a promising medicinal plant candidate with significant inhibitory effects on multidrug resistance therapy [51]. Also, *P. polyphylla* saponins have broad-spectrum antimicrobial effects, including acne-causing bacteria [23], such as *P. acnes*, *S. aureus*, *S. epidermidis*, etc. Therefore, *P. polyphylla* can be used as an effective medicinal plant for treating acne and reducing drug resistance.

### 3.2. Inflammatory Reaction

As a primary pathogen causing acne, the overgrowth of *P. acnes* affects humans and triggers a severe inflammatory response. The body recognizes microbial components through pattern recognition receptors (PRRS) of the innate immune system to protect the host from infection. The type of pattern recognition receptors associated with acne is the toll-like receptors (TLRs). TLRs are evolutionarily conserved transmembrane innate immune receptors involved in the first line of defense of human health and play important roles in recognizing pathogen-associated molecular patterns (PAMPs) [52]. The active TLR2 is expressed in skin cells [53], such as keratinocytes, monocytes, and macrophages, and is activated by binding to Acinetobacter peptidoglycan, promoting the production of inflammatory factors (e.g., IL-8, IL-6, IL-1β, TNF-α) [54], and stimulating the differentiation of monocytes into macrophages. 

Furthermore, it can activate the MAPK signaling pathway. By triggering protein phosphorylation of ERK, JNK, and P38 protein kinases [55], the expression of inflammatory proteins (iNOS, COX-2) and caspase-8 and 9 productions are promoted [56,57], thereby activating the production of NO and PGE2 inflammatory mediators. 

It was found that prolonged exposure to inflammation increases the levels of inflammatory factors in hair follicles and perifollicular vessels, including human leukocyte antigen-DR (HLADR), intercellular adhesion molecule-1 (ICAM-1), and vascular cell adhesion molecule-1 (VCAM-1), in the perifollicular vessels [58]. Expressing adhesion molecules in the vascular endothelium surrounding the sebaceous ducts causes the accumulation of lymphocytes and neutrophils in the peripheral blood, leading to an immune response and inflammation around the hair follicle [59]. Acne can also induce a specific response of CD4^+^T cells and promote the differentiation of T cells into Th17 and Th1 cells [60], promoting the production of inflammatory factors (such as IL-17 [61] and IL-4 [62]). Karadag et al. [62] found that serum Th1/Th2 cytokine levels were imbalanced in severe acne, with decreased levels of interferon-gamma (IFN-γ).

Due to their potent anti-inflammatory effects and ability to modulate the immune system, *P. polyphylla* saponins can be used as a safe and efficient phytoconstituent for treating acne.

### 3.3. Sebum Secretion

Excessive sebum production aggravates acne. Excess sebum leads to follicle blockage, creating an anaerobic environment that provides a suitable condition for the growth of Acinetobacter, which further interferes with the skin barrier and promotes the production of seborrheic acne. Androgens in the sebaceous glands regulate the maturation and secretion of sebaceous glands and thus stimulate the synthesis, proliferation, and differentiation of adipocytes [63]. Androgen receptors bind to dihydrotestosterone (DHT) [64] by stimulating the PI3K/Akt/mTOR cascade [65], thereby activating the expression of sterol regulatory element binding protein-1 (SREBP-1) and accelerating lipid synthesis [66,67].

The PI3K/Akt signaling pathway is closely linked to acne sebum metabolism and can reduce lipogenesis (including cholesterol, triglycerides, and free fatty acids) in human sebocytes [68]. Insulin-like growth factor 1 (IGF-1) regulates the PI3K/Akt/mTOR signaling pathway by binding to the insulin-like growth factor 1 receptor (IGF-1R) and decreases the transcriptional activity of the recombinant forkhead box protein O1 (FoxO1) in SZ95 sebaceous gland cells thereby inducing lipid synthesis [69]. Meanwhile, IGF-1 can also promote the production of MMPs (such as MMP-2 and MMP-9) by activating the NF-κB pathway, which is linked with lipogenesis promotion [70].

The Wnt/β-catenin signaling pathway negatively regulates adipogenesis. Androgens can induce sebocyte differentiation and cause excessive sebum production by inhibiting Wnt/β-catenin signaling [71]. Although there is experimental evidence that *P. polyphylla* saponins can regulate signaling in the PI3K/Akt [72] and Wnt/β-catenin [73] signaling pathways, the mechanism of the anti-acne action still needs further study.

### 3.4. Hyperkeratosis

Dyskeratosis of the basal layer and abnormal activities of keratinocytes in the ducts play parts in acne occurrence and development. Excessive keratinocyte proliferation thickens the epithelial layer of the sebaceous ducts and reduces their diameter, eventually leading to acute occlusion of the sebaceous ducts and thin-walled cystic lesions (acne).

Keratins are intermediate filament proteins that connect the cytoskeleton of keratinocytes and have an essential role in matrix rigidity sensing and downstream signaling [74]. Keratins are sometimes expressed in a pairwise manner, with a type I keratin (acidic, keratins 9–20) paired with its specific type II partner (basic, keratins 1–8), for example, K6/K16 and K1/K10 [75]. Generally, K1/K10 are expressed in healthy individuals, while K6/K16 are expressed in acne patients [76,77]. The binding of androgens and androgen receptors on keratin-forming cells inhibits the expression of the normal K1/K10 in the basal layer [78], thereby promoting the proliferation of keratin-forming cells arranged in the hair follicle portion of the sebaceous glandular unit of the hair follicle. At the same time, keratinocytes can interact with *P. acne* to enter the dermis, stimulate an inflammatory cascade response that leads to impaired skin barrier function, and release pro-inflammatory factors, such as IL-1 α and TNF-α [77]. The secretion of IL-1α stimulates keratin-forming cell activation and promotes acne by inducing the synthesis of keratin 6 in epidermal keratin-forming cells [79]. *P. polyphylla* saponins have been found to regulate the expression levels of keratin and the inflammatory factors IL-1α and TNF-α [37]. Thus, they can be a promising treatment for acne problems caused by hyperkeratosis.

## 4. Bioactives of *P. polyphylla* Associated with Acne Treatment

The study of the chemical composition of *P. polyphylla* can be traced back to 1938. Dutt [80] separated paride and paristyphnin from *P. quadrifolia* L. Then, in 1962, Huang et al. [81]. isolated diosgenin and pennogenin from the rhizome of *Polyphylla* var. *yunnanensi*. Since then, scholars all over the world have isolated about 67 steroidal saponins from 11 species of plants in the genus Paris [82] and have identified 323 compounds respectively [39], including steroidal saponins, phytosterols [83,84,85], polysaccharides [86], triterpenoids [87,88], flavonoids [89,90] and other chemical components [91,92,93]. Steroidal saponins are the primary compounds in *P. polyphylla*, accounting for about 52% of the total chemical components [39]. Among them, more than 50 species were identified from *P. polyphylla* var. *yunnanensis* [94]. Importantly, *P. polyphylla* saponins I, II, VI, and VII have been recognized as the quality standard components in the Chinensis Pharmacopoeia [14].

**Figure 4:**
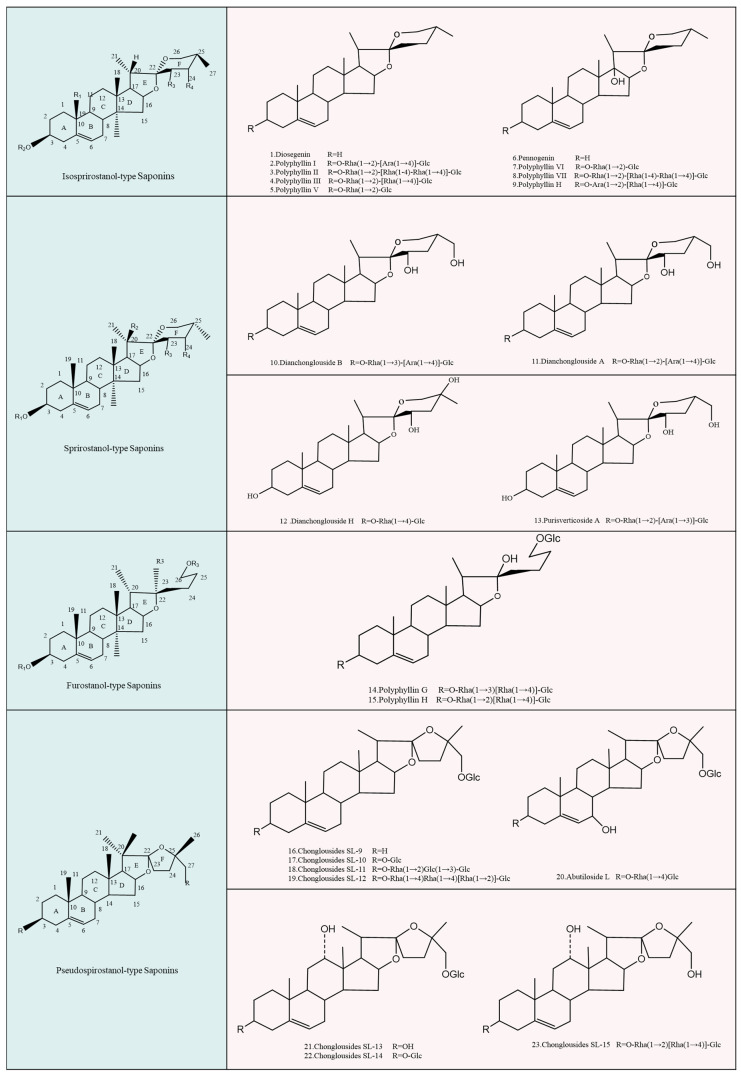
Active ingredients of *P. polyphylla* saponin. *P. polyphylla* saponin can be divided into four main groups, namely isospirostanols, spirostanols, furostanols, and pseudospirostanols, and typical monomers are listed.

Steroid saponins are an important research target for treating acne due to their high pharmacological activities, such as their anti-inflammatory, antibacterial, and immune system-modulating effects. Steroid saponins are glycosides consisting of a hydrophobic glycoside element (C3-linked steroidal saponins or triterpenoids) [95] and one or more hydrophilic sugar groups (glucose, galactose, pentose, or methyl pentose) [96]. They are classified into four major groups by the conformation of C25, the cyclization state of the F ring, and the spiroalkane structure [97]. Figure 4 describes the four groups, namely isospirostanols, spirostanols, furostanols, and pseudospirostanols [98]. In addition, we analyzed and compared their structural characteristics and listed the typical monomers.

### 4.1. Isospirostanol Type

Most of the glycosides are diosgenin and pennogenin, containing a double bond at the 5(6) position and hydroxyl substituents at the 3β, 7β, 23β, 24β, and 27β positions, where the sugars are linked to the hydroxyl substituents to form saponins. The sugar fraction consists of D-glucose, L-rhamnose, L-arabinose, and to a lesser extent, D-xylose and L-fructose. The sugar group forms glycosides with the glycoside C3-OH and, to a lesser extent, with C1-OH, C21-OH, C23-OH, C26-OH, and C27-OH.

The bioactive compounds with potential acne treatment mainly belong to the isospirostanol type. They include diosgenin [81] (No. 1 in Figure 4), pennogenin [97] (No. 7), PP I (No. 2), Polyphyllin II (PP II, No. 3), Polyphyllin III (PP III, No. 4) [99], Polyphyllin V (PP V, No. 5), Polyphyllin VI [98] (PP VI, No. 8), Polyphyllin VII [8] (PP VII, No. 9) and Paris saponins H [8] (No. 10).

### 4.2. Spirostanol Type

Spirostanol type is a class of steroidal saponins with C25 as S-configuration. It is the epimer of the isospirostanol type [100,101]. Seven major spirostanols can be extracted and isolated from *P. polyphylla* [40], primarily dianchonglouoside A (No. 12 in Figure 4), dianchonglouoside B (No. 11), disoseptemloside H (No. 13) [9] and parisverticoside A [25] (No. 14), etc.

Currently, there is limited literature about compounds of this type for treating acne. Therefore, further studies are needed to explore the possibility of acne treatment or the causality of the potential associations.

### 4.3. Furostanol Type

Furostanol-type saponins are a class of F-ring cleavage compounds. The hydroxyl substituents are at the 3, 7, 17, and 26 positions and form steroidal saponins in addition to saponins linked to the sugar group at C3-OH and glucose at C26-OH to form a double sugar chain. Solvents containing -CH3 (e.g., methanol) are avoided during separation as their C22-OH is susceptible to substitution to form OCH_3_ products. Plant enzymes readily metabolize their C26-OH sugar chain to close the ring with C22-OH dehydration to form the corresponding spirostanol precursor compound. Thus, the furostanol-type steroid saponins are often considered precursors of the spirostanol-type steroidal saponins [25,102].

Saponins belonging to this type include polyphyllin G (PP G No. 15 in Figure 4) and polyphyllin H (PP H, No. 16) [102]. However, due to the instability of the F-ring in the furostanol-type, it is easily cleaved into a double sugar chain saponin, resulting in a lack of biological activity such as hemolysis, antibacterial action and cytotoxicity, and even difficulty in forming complexes with cholesterol [103]. As a result, selecting this saponin as the active ingredient in acne treatment is complex.

### 4.4. Pseudospirostanol Type

The pseudospirostanols type is an F-ring open chain structure containing a five-membered tetrahydrofuran ring. This type of component can be isolated and identified only from the stem and leaves of *P. polyphylla* var. *yunnanensis*, with a total of 13 species [104]. Liu et al. [98] obtained pseudospirostanol–type compounds, including chonglouside SL-9–chonglouside SL-15 (No. 16–19; No. 21–23 in Figure 4) and abutiloside L (No. 20 in Figure 4), which have strong antibacterial effects and possibilities to be used as active antibacterial substances for the treatment of acne.

## 5. *P. polyphylla* Saponins Play Roles in Acne Treatment

Some key features of acne development include disturbed sebaceous gland activity resulting in excessive sebum, altered sebocyte proliferation and differentiation, dysregulation of the hormonal environment, hyperkeratinization, colonization of *P. acnes*, and inflammation [105,106]. Therefore, the roles related to acne treatment of *P. polyphylla* saponins may include anti-inflammatory, antibacterial, immunomodulatory, and antioxidant effects. In addition, sebaceous gland function regulation has also been summarized (Figure 3).

### 5.1. Anti-Inflammatory Effects

The anti-inflammatory effects of *P. polyphylla* saponins have been widely reported. They demonstrate cytokine regulatory effects and a close relationship between anti-inflammation and acne treatment. The main saponins showing anti-inflammatory effects include Rhizoma Paridis total saponins and some monomers, such as PP I, PP II, PP VI, PP VII, and diosgenin.

*Paris polyphylla* saponins show multiple anti-inflammatory mechanisms. They exert anti-inflammatory effects in a dose-dependent manner. It effectively inhibits the release of pro-inflammatory factors, such as TNF-α [37,107], IL-1β [28,37,108], CXCL16 [8], IL-1α, IL-6, IL-8 [37], IL-17, IL-23 [109], NO [110], PGE2, iNOS, COX-2 [108], etc. The antioxidant enzymes, such as superoxide dismutase (SOD) and CAT, are also increased, thus scavenging free radicals and decreasing the peroxidation products in the inflammatory models [38]. Furthermore, the matrix metalloproteinases, such as MMP-2 [111], MMP-3 [108], MMP-9 [111], and MMP-13 [108], are down-regulated by *P. polyphylla* saponins, indicating their excellent recovery effects against inflammation. Table 1 lists the latest results describing anti-inflammatory studies of *P. polyphylla* Saponins from the literature published between 2012 and 2022.

Man et al. [8] found that *P. polyphylla* saponins can modulate cytokines or receptors such as VEGFD, VEGFR3, RAGE, IL-6R, IL-17BR, and CXCL16, and increase the levels of SOD and CAT enzymes [38]. *P. polyphylla* saponins can also inhibit the abnormally active NF-κB and reduce PI3K/Akt and MAPK (including p38, ERK1/2, and JNK) phosphorylation [8]. The inhibition of NO release in lipopolysaccharide (LPS)-induced RAW264.7 mouse macrophages were observed after treating Rhizoma Paridis total saponins, and there was no significant cytotoxicity [38,110]. According to the structural diversity, many researchers investigated the saponin monomers’ anti-inflammatory abilities in different diseases. PP I and PP VII are the most studied and widely used in current medical practice among the identified saponin monomers.

PP I is one of the saponins, showing anti-inflammatory effects and playing a part in acne treatment. Yang et al. [28] found that PP I could inhibit *P. acnes*-induced excessive proliferation and abnormal differentiation of HaCaT cells. It downregulated the expressions of CD36, NOX1, NLRP3, and Asc, decreased caspase-1 activity, and decreased the secretion of IL-1β and IL-8. Meanwhile, ROS production in HaCaT cells was also down-regulated. Moreover, according to Zhu et al. [37], p38 phosphorylation is inhibited by PP I in a dose-dependent manner, with decreased IL-6, IL-8, TNF-α, and TLR2 expressions. Generally, the acceleration of the TLR2 pathway is correlated with increased iNOS and COX-2 expression and cytokine secretion [56]. In addition to tIL-1α mentioned above, IL-1β, IL-6, IL-8, and TNF-α, IL-17 and IL-23 are key pro-inflammatory cytokines. PP I could down-regulate the expression of VEGF and IL-23 in HaCaT cells in a dose-dependent manner, thereby inhibiting the secretion of IL-17 [109]. Another study also found that PP I could down-regulate the expression of K16 in heat-inactivated Acinetobacter-treated HaCaT cells [37]. Meanwhile, Zhu et al. [37] found that PP I contributes to the regulation of cytokine gene expression by inhibiting the PI3K/Akt/mTOR signaling pathway and NF-κB-mediated production of pro-inflammatory factors in activated macrophages, thus providing a new therapeutic target for PP I to control inflammatory acne.

Zhang et al. [112] verified the anti-inflammatory effects of PP II in vivo, using mouse and zebrafish models separately. It was found that PP II could effectively inhibit the abnormally active NF-κB pathway and reduce the production of MMP-9, thereby reducing VEGF production and decreasing the inflammatory response through anti-angiogenesis.

PP VII is an isospirostanol saponin with anti-inflammatory properties that inhibits angiogenesis, lymph angiogenesis, adhesion, inflammation, and invasiveness. In a study, PP VII inhibited NO and PGE_2_ production stimulated by LPS in the model of zebrafish embryos. In the PP VII group, the biomarkers iNOS, COX-2, and MMP-9 were inhibited in LPS-induced RAW264.7 cells. NF-κB and MAPKs played parts in the research [26]. Meanwhile, another study [27] proved the decreased tendencies of the protein expressions of PI3K, (p)-PI3K, Akt, p-Akt, NF-κB, and p-NF-κB after PP VII treatment. Thus, it might be concluded that PP VII can reduce inflammatory damage by affecting NF-κB, MAPKs, and PI3K/Akt pathways, which could be the targets for the subsequent treatment of inflammatory acne with PP VII.

Diosgenin plays a vital role in anti-inflammatory effects. Pro-inflammatory factors were detected in low levels after treatment with diosgenin. It was demonstrated that diosgenin inhibited IL-1β-induced NO and PGE_2_ production and significantly decreased the expression of MMPs (MMP-3 and MMP-13), iNOS and COX-2 in IL-1β-stimulated human OA chondrocytes [108]. Furthermore, some factors related to adhesion, such as VCAM-1 and ICAM-1, were inhibited by diosgenin [113]. Choi et al. [113] found that such factors can inhibit TNF-α-induced THP-1 monocyte adhesion and decrease the expression of VCAM-1 and ICAM-1 in vivo. It was reported that the phosphorylation of p38, ERK, JNK, and Akt played parts in the diosgenin process [113]. It can also decrease the production of ROS [114].

Another type of saponin named PP H was reported to have similar effects with to dexamethasone in improving bacterial-infected inflammatory skin equivalents [115]. In the study, a biomimetic “interface-controlled-skin-on-chip” system was constructed. *P. acnes* and sodium lauryl sulfate stimulation were performed to damage the barrier function. After the treatment with PP H, a significant repair effect on the skin barrier and inhibition on the re-lease of inflammation-related cytokines were observed. Fuethermore, the effects were more prominent than those with dexamethasone. According to the study by Yang et al. [116], the in-depth mechanism may be that PP H inhibited the nuclear translocation of NF-κB P65. PP H could bind to Keap1 and activate Nrf2, thus upregulated HO-1 in LPS-induced RAW 264.7 cells, based on a molecular docking study.

### 5.2. Anti-Bacterial Effects

*P. acnes* biofilms were reported to be more frequent in acne lesions than in control follicles [117]. *P. polyphylla* saponins have broad-spectrum antibacterial and antifungal activities (Table 1). It was reported that they showed potent inhibition against Gram-positive and Gram-negative bacteria with MIC values ranging from 13.1 to 78 μg/mL and fungi with an antibacterial rate from 8.32% to 56.50% [118]. Among them, *P. acnes*, *S. aureus*, and *S. epidermidis* were strongly inhibited [23]. Accordingly, they showed strong inhibition to *S. aureus* ATCC29213 (MIC = 12.2 μg/mL, MBC = 24.4 μg/mL) and *S. epidermidis* CMCC260 (MIC = 48. 7 μg/mL, MBC = 97. 5 μg/mL) [70] and showed weak inhibition against *P. acnes* NCTC737L and *P. acnes* ATCC6919 (MIC = 97.5 μg/mL, 48.7 μg/mL; MBC = 198 μg/mL, 97.5 μg/mL, respectively) [119].

Among the identified saponin monomers (PP I, PP II, PP VI, PP H, and PP VII), PP I has the strongest antibacterial effect, while PP H has a relatively weak antibacterial effect [120].

Scholars compared the antimicrobial abilities between the *P. polyphylla* saponins and antibiotics and found different phenomena. For example, Sun et al. [23] found that *P. polyphylla* saponins were slightly lower in ability than antibiotics, while in the study of Wang et al. [119], the conclusion was drawn that the MIC values for the antimicrobial activities of *P. polyphylla* saponins were higher than those expressed by antibiotics.

Undoubtedly, the antibacterial strength against different pathogens varies because of the different structures of *P. polyphylla* saponins. It was reported that the glycosyl groups and lengths of sugar chain linkages in the structure of saponins affect the antibacterial effect [9]. According to Qin et al. [9], Chonglouoside SL-6, containing a trisaccharide sugar moiety at C1, exhibited the greatest most antimicrobial effect among the different saponin structures. In contrast, PP V and dioscin containing hydroxyl derivatives at C7 or C25 showed diminished activity against *P. acnes*.

As a result, the relationships between the chemical structure and the antibacterial capacity of *P. polyphylla* saponins require further investigation, potentially providing new ideas for developing acne-related drugs in the future.

### 5.3. Immunomodulatory Effects

The activation of immune signaling pathways can be a double-edged sword, as this is necessary to clear pathogens but harmful when too sustained or uncontrolled. For example, a recent report highlights that the persistence of inflammation in acne lesions is linked to prolonged lesions, scar formation, and loss of the sebaceous gland [121]. This suggests that, apart from with anti-inflammatory treatments, moderate immunomodulation is necessary for acne.

In the study, many CD4^+^T cells in acne patients differentiated into Th1 and Th17 cells, which increased IL-17 and IFN-γ [10]. *P. polyphylla* saponins could inhibit CD4^+^T cell proliferation and hinder Th1/Th17 cell differentiation [109,114,122,123], decreasing IL-4 and increasing IFN-γ production [109], thus improving the immunosuppressive function of CD4^+^T cells. The increase in serum interferon (IFN-γ) and IL-2 levels and the decrease in IL-4 levels were also observed, resulting in a shift in the Th1/Th2 balance towards Th1 [123].

VCAM-1 and ICAM-1 are glycoproteins on the cell surface and are members of the immunoglobulin superfamily. Stimulating vascular endothelial cells by inflammatory factors promotes the interaction of VCAM-1 and ICAM-1 with ligands, resulting in the aggregation of inflammatory macrophages, immunomodulation, platelet aggregation, and adhesion [124,125,126]. The anti-angiogenesis of *P. polyphylla* saponins and the inhibited expression of VEGF-1 and ICAM-1 finally lead to immunomodulation. Chai et al. [127] found that *P. polyphylla* saponins could effectively reduce the expression of VEGF-A, VCAM-1, IL6R, IL17BR, and CXCL16 in a mouse model of lung adenocarcinoma. Furthermore, the expression levels of ICAM-1 and VCAM-1 mRNA were inhibited in response to oxidative stress [128]. The reduced expression of HIF-1α and VEGF was also observed after the intervention of PP I in hypoxic laryngeal carcinoma Hep-2 cells [109]. However, current studies on the mechanisms of *P. polyphylla* saponins in immunomodulation are mainly focused on cancer. In the future, we hope to research the relationship between *P. polyphylla* saponins and immunomodulatory-related mechanisms in acne pathogenesis.

### 5.4. Sebaceous Gland Function-Regulating Effects

Sebum is a waxy, lipid-rich biofluid excreted by the skin’s sebaceous glands, and its overproduction is a known reason for acne. Increased sebum excretion is a major factor in the pathophysiology of acne. Other sebaceous gland functions include the regulation of cutaneous steroidogenesis, local androgen synthesis, and hormonal control [129]. Abnormal sebaceous gland function tends to cause disturbances in lipid metabolism and increased lipid synthesis.

Although none of the literature shows direct relationships between *P. polyphylla* saponins and lipid synthesis, Rhizoma Paridis total saponins were found to inhibit the PI3K/Akt and MAPK (p38, Erk1/2, JNK) signaling pathway [130,131]. Liu et al. [132] demonstrated that inhibition of the human sebocyte PI3K/Akt signaling pathway is associated with decreased lipogenesis (including cholesterol, triglycerides, and free fatty acids). Furthermore, the androgens can promote sebaceous gland hyperplasia by inhibiting the Wnt signaling pathway, while PP VII was reported to promote this pathway [133], which is further indicated to have the potential to reduce lipid synthesis. Therefore, further research is needed to explore the role of *P. polyphylla* saponins in the pathogenesis of seborrheic acne.

### 5.5. Follicular Hyperkeratosis Effects

Hyperkeratosis and hyperproliferation of funnel keratinocytes in acne are accompanied by overexpression of K6 and K16 [37]. It was found that stimulating androgen-mediated FGFR2 signaling [134] and increasing IL-1α expression [58] could increase the occurrence of acne. IL-1α subsequently activates basal keratin-forming cells by induction of K16 expression by autocrine products. Zhu et al. [37] found that K6 and K16 are detected in large amounts in the lesions of acne patients compared to the skin surface of healthy individuals.

It was reported that PP I could modulate keratin expression, inhibit IL-1α release, and suppress K16 expression in HaCaT keratinocytes treated by heat-inactivated P. acne [37]. Thus, PP I may be used as a health therapy to improve seborrheic acne.

### 5.6. Antioxidant Effects

Excessive ROS can lead to oxidative damage to cellular lipoproteins, proteins, and DNA, thus resulting in inflammatory responses that promote acne, photooxidation, and other skin problems [135]. In addition, the unsaturated fatty acids cause lipid peroxidation in lipids undergoing auto-oxidation to produce unstable hydroperoxides. The resulting hydroperoxides continue to decompose to form small molecules of compounds such as acids, aldehydes, and ketones with short carbon chains [136,137]. Ayres et al. [138] found that acne patients develop lipid peroxidation in their bodies, leading to the progression of acne conditions.

Due to its complex structure, the chemical structure of *P. polyphylla* saponins contains phenolic hydroxyl groups, which can terminate free radical chain (·OH and O^−2^) reactions by binding to free radicals to form stable semi-keto radical structures [139]. Secondly, the phenolic hydroxyl structure prevents the production of hydroxyl radicals by chelating Fe^2+^, Cu^2+^, and other trace elements in the system [140,141], thus reducing the content of reactive oxygen species. Finally, *P. polyphylla* saponins can reduce free radical activity by forming hydrogen bonds as a type of hydrogen donor. Comparison of the antioxidant capacity of PP VII and ascorbic acid (VC) at the same concentration has been reported to show that VC has a stronger scavenging capacity for ·OH and O^−2^ than PP VII, but the scavenging capacity of PP VII for DPPH was stronger than that of VC at concentrations higher than 4 mg/mL [142].

Furthermore, *P. polyphylla* extracts can act as antioxidants with protective effects against intracellular oxidative stress. Gao et al. [143] found that PP I reduced oxidative stress by activating the SIRT3/SOD2/ROS signaling pathway. Das et al. [38] further evaluated the antioxidant activities in vitro and found that diosgenin increased the intracellular nitric oxide dismutase (NOD) and SOD. Meanwhile, *P. polyphylla* saponins were also proved to have the ability to resist lipid oxidation and protect DNA, thus indirectly exerting its protective effect [140] in the model of dextran-induced rat paw edema.

The clinical studies of P. polyphylla extracts on anti-acne effect were in a minority. Xu et al. [144] reported the clinical observation of a skin care product containing the extract of many leaf Paris rhizome and purslane. The acne effect was observed in the treatment of acne vulgaris. In the study of Fang Ting, a hydrogel containing *P. polyphylla* ex-tract for the treatment of acne was produced. The preliminary quality research and safety evaluation of that hydrogel were performed and obtained good results, but no clinical trial was designed [145]. 

**Table 1:** Biological activities and their mechanisms of Paris saponins.

No.		Substance	Research Subject	Evaluation	Cytokines	References
1	Anti-inflammatory effects	PP I	HACAT cells induced by *P. acnes*	ELISA PCR/Western blot	↓ CD36/NOX1/ROS/NLRP3/IL-1β Pathway, IL-8.	[28]
2			HACAT cells induced by *P. acnes*	ELISA PCR/Western blot	↓ IL-6, IL-8, TNF-α, ↓ NF-κB activation, p38 phosphorylation, TLR2 expression.	[37]
3			LPS and IFN-γ induced primary bone marrow-derived macrophages (BMMs) and peritoneal elucidated macrophages (PEMs) cell model in mice	PCR/Western blot	↓ NF-κB-mediated production of pro-inflammatory effectors in activated macrophages.	[146]
4			IL-17-stimulated HaCaT cell model	CCK-8/PCR	↓ IL-17 stimulated VEGF, IL-23 content and VEGF mRNA, IL-23 mRNA expression.	[109]
5		PP VII	LPS-induced RAW264.7 cell model of male/zebrafish	ELISA/PCR/Western blot	↓ NO and PGE_2_ production as well as pro-inflammatory cytokines (TNF-α, IL-1β and IL-6), enzymes (iNOS, COX-2), MMP-9 protein and mRNA expression.	[26]
6			HeLa cells, A549 cells, HepG2 cells	MTT/Western blot	↓ NF-κB/MMP-9/VEGF pathway.	[112]
7			Induction of apoptosis in an A549 human lung cancer cells model	phase-contrast microscopy/fluorescence microscopy/flow cytometry/Western blot analysis.	↓ PI3K/Akt and NF-κB pathways.	[27]
8		PP VI	LOVO cell model of intestinal cancer induction	Western blot	↓ MMP-2, MMP-9 expression.	[111]
9		*Rhizoma Paridis* total saponins	LPS-induced murine macrophage (RAW 264.7) model	CCK8/Griess	↓ NO release amount.	[110]
10			Heat-inactivated *Escherichia coli*-induced macrophage model in rat peritoneal cavity	ELISA	↓ TNF-α, IL-1β.	[107]
11			Lewis lung adenoma mouse model of induced lung adenocarcinoma cells	PCR/Western blot	↓ VEGFD, VEGFR3, RAGE, IL6R, IL17BR and CXCL16, ↑ SOD and CAT, phosphorylation of NF-κB, PI3K/Akt, MAPK (p38, Erk1/2, JNK) signaling pathway.	[8]
12			Dextran induced hind paw edema in rats	MTT/fluorescence microscopy/PCR	Demonstrated potent anti-inflammatory activity by dose-dependently inhibiting dextran-induced paw edema in rats (*p* < 0.01) over a period of 2 h to 4 h.	[38]
13		Diosgenin	Osteoarthritis (OA)-induced human OA chondrocytes	ELISA	↓ IL-1β, NO, PGE_2_ generation, ↓ MMP-3, MMP-13, iNOS, COX-2 expression.	[108]
14			A molecular model of TNF-α-induced adhesion in the mouse VSMC cell line MOVAS-1	ELISA/PCR/Western blot	↓ VCAM-1 and ICAM-1 mRNA and content expression, ↓ ROS, ↓ p38, ERK, JNK and Akt phosphorylation, ↓ NK-κB activation.	[113]
15		PP II	Human ovarian cancer cell-induced angiogenesis model	MTT/EMSA/Western blot	↓ NF-κB activity and VEGF-mediated angiogenesis.	[147]
16			Primary liver cancer-induced HepG2 and BEL7402 cell model	ELISA/PCR/Western blot	↓ NF-κB activity andMMP2/MMP9 mRNA and content expression.	[148]
17		PP H	Lipopolysaccharide (LPS)-induced RAW 264.7 and HaCaT cells	ELISA/PCR/Western blot/molecular docking/surface plasmon resonance analysis	↑ NRF2/HO-1 antioxidant pathway, ↓ activation of the MAPK pathway, ↓ the nuclear translocation of NF-κB and downstream inflammatory genes expression.	[116]
18	Antibacterial effects	PP G	Human oral cancer induced Gram-positive and Gram-negative bacteria	The broth microdilution method	Gram-positive and Gram-negative bacteria (MICs = 13.1–78 µg/mL).	[118]
19		Chonglouoside SL-7	Positive control: erythromycin	The broth microdilution method	Antibacterial (MIC = 31.3, 3.9 μg/mL).	[149]
20		*Rhizoma Paridis* total saponins (PP I, II, III, V, VI, VII, H)	*P. acnes* NCTC737, ATCC6919, *S. epidermidis* ATCC12228, *S. aureus* ATCC6538/positive control: erythromycin, clindamycin	Liquid microdilution method	Rhizoma Paridis total saponins inhibits *P. acnes* NCTC737 and ATCC6919, *S. epidermidis* ATCC12228, *S. aureus* ATCC6538 (MIC = 2.5, 5.0, 5.0, 1.25 mg/mL), Polyphyllin I, II, III, V, VI, VII, H inhibits *P. acnes* NCTC737 (MIC = 0.6, 1.2, 2.5, 5.0, 2.5, 2.5, 10.0 mg/mL), *S. epidermidis* ATCC12228 (MIC = 1.2, 1.2, 5.0, 5.0, 2.5, 2.5, 10.0 mg/mL), *S. aureus* ATCC6538 (MIC = 0.6, 0.6, 2.5, 5.0, 2.5, 2.5, 10.0 mg/mL).	[120]
21		*Rhizoma Paridis* total saponins	*P. acnes* NCTC737, ATCC6919, *S. epidermidis* CMCC26069, *S. aureus* ATCC29213/positive control: erythromycin, clindamycin	Agar dilution method/broth microdilution method	*P. acnes* NCTC737 (MIC = 97.5 μg/mL, MBC = 198.0 μg/mL), *P. acnes* ATCC6919 (MIC = 48.7 μg/mL, MBC = 97.5 μg/mL), *S. epidermidis* CMCC26069 (MIC = 48.7 μg/mL, MBC = 97.5 μg/mL), *S. aureus* ATCC29213 (MIC = 12.2 μg/mL, MBC = 24.4 μg/mL), erythromycin inhibits *P. acnes* NCTC737, ATCC6919 (MIC = 0.0625 μg/mL), *S. epidermidis* CMCC26069 (MIC = 7.8 μg/mL), *S. aureus* ATCC29213 (MIC = 0.0625 μg/mL), clindamycin inhibits *P. acnes* NCTC737 (MIC = 0.125 μg/mL), *P. acnes* ATCC6919 (MIC = 0.0625 μg/mL).	[23]
22		PP I, PP II, PP VI, PP VII		Liquid microdilution method	Polyphyllin I, II, VI, VII inhibition *P. acnes* (MIC = 125 μg/mL), *S. epidermidis* (MIC = 15.6, 15.6, 500, 31.2 μg/mL), *S. aureus* (MIC = 15.6, 15.6, 500, 15.6 μg/mL).	[119]
23	Immunoregulation Sebum secretion	*Rhizoma Paridis* total saponins	Mouse asthma model	ELISA/PCR	↓ Th1/Th2, ↓ IL-4 and IFN-γ.	[150]
24			Polytrauma rat model	ELISA	↑ TNF-ɑ, IL-1 and IL-6.	[151]
25			Lupus Nephritis-induced lymphocytes model	MTT/ELISA	Regulates Th1/Th2 imbalance and enhances immunosuppressive function of CD4^+^Treg and CD25^+^Treg.	[122]
26			Lewis lung adenoma mouse model of induced lung adenocarcinoma cells	PCR/Western blot	↓ VEGFD, VEGFR3, RAGE, IL6R, IL17BR and CXCL16, ↑ SOD, catalase enzyme content,	[8]
27		Diosgenin	Multiple sclerosis-induced microglia and macrophages modellerosis	Fluorescence microscopy	↓ microglia and macrophages activation, ↓ CD4^+^T cell proliferation, ↓ Th1/Th17 cell differentiation.	[114]
28		PP I		ELISA/PCR/Western blot	↓ HIF-1α, VEGF.	[109]
29	Follicular hyperkeratosis effects	PP I	Acne caused by *P. acnes*	PCR/Western blot	↓ IL 1α and K16 expression levels in HaCaT keratin cells.	[37]
30	Anti-oxidation	*Rhizoma Paridis* total saponins	Ascorbic acid	MTT/AO/PI staining/fluorescence microscopy/PCR	↑DPPH, NOD, SOD, RP.	[38]
31		PP I	HacaT photoaging model caused by UV light	ELISA/WB/DCFH-DA	↑ SIRT3, SOD2, ↓ p53acetylation levels, Bax, cleaved caspase3, ROS.	[143]
32		PP II	Glomerular mesangial cells under high glucose intervention (GMC)	MTT/DCFH-DA/xanthine oxidase assay/malondialdehyde kit	↓ MDA, ROS, ↑ SOD.	[114]
33		*Rhizoma Paridis* total saponins	Lipid peroxidation model induced by light riboflavin and the Fenton reaction	Spectrophotometric methods	Scavenging ·OH, O-2 radicals, ↓ lipid peroxidation and ·OH induced oxidative DNA damage.	[140]

Note: ↓ for inhibition, ↑ for promotion.

## 6. Extraction Methods of *P. polyphylla* Saponins

There are various extraction processes for saponins, such as reflux extraction (RE), ultrasonic-assisted solvent extraction (USA-SE), microwave-assisted solvent extraction (MWA-SE), ultra-high-pressure-assisted solvent extraction (UHPA-SE), supercritical fluid CO_2_ extraction (SFE-CO_2_), and aqueous enzymatic extraction (AEE).

**Figure 5:**
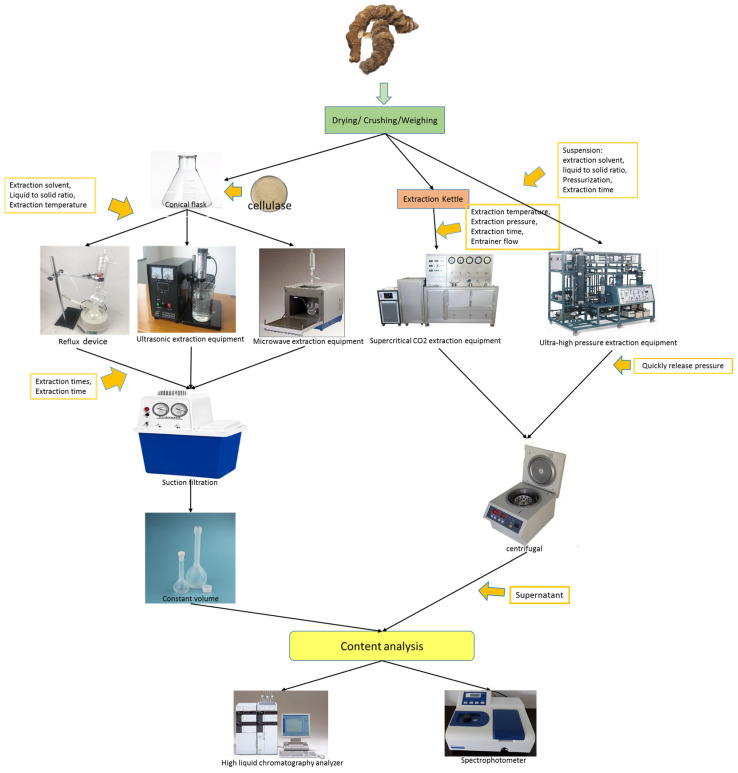
Extraction processes of *P. polyphylla* saponins. The extraction process of *P. polyphylla* saponins consists of reflux extraction (RE), ultrasonic-assisted solvent extraction (USA-SE), ultra-high-press-sure-assisted solvent extraction (UHPA-SE), microwave-assisted solvent extraction (MWA-SE), and supercritical fluid CO_2_ extraction (SFE-CO_2_). The diagram shows the different extraction processes using different extraction equipment, by adjusting the reaction conditions for the extraction of *P. polyphylla* saponins.

In the study, a summary of the standard extraction techniques has been made to provide helpful information for the relevant processing industries. Figure 5 clearly shows the similarities and differences between the different extraction processes. We also described some extraction conditions of *P. polyphylla* saponins in Table 2. Furthermore, Table 2 outlines the extraction techniques according to the objectives and further indicates the advantages and disadvantages of the different extraction treatments to provide a basis for the later selection of extraction methods. The detailed description of these extraction methods is also shown in the Supplementary Materials attached to the manuscript.

**Table 2:** Paris extraction processes reported in literature.

No.	Extraction Method	Extraction Components	Extraction Conditions	Detection Method	Optimal Process Conditions	Rate	References
1	RE	Diosgenin	Extraction solvent: 75~95% ethanol, solvent dosage: 4~8 mL, extraction time: (0.5, 0.5)–(1.5, 1.5) h, extraction times: 2.	HPLC/orthogonal experimental method	The crude powder of Paris herb was refluxed twice with 8 times the amount of 85% ethanol, 1.5 h/time each. The order of effect was as follows: reflux time > ethanol concentration > ethanol dosage	The average content of diosgenin element was 6.0821 mg/g.	[152]
2	RE	Rhizoma Paridis total saponins/ PP I/PP II	Extraction solvent: water, 30–90% ethanol, particle size of herbs: drinking tablets, coarsest powder, coarse powder, solvent dosage (first time, second time): 1:(6, 4)–1:(10, 8) (g:mL), extraction time: (1, 0.5)–(2, 1.5) h, extraction times:1~3.	HPLC method	70% ethanol was extracted twice, the first time with 10 times the amount for 2 h, the second time with 8 times the amount for 1.5 h. The order of effect size was as follows: reflux time > ethanol dosage > ethanol concentration. The effect of the crushing degree of herbs on the total saponin yield was small.	The *Rhizoma Paridis* total saponins yield was 4.24%, RSD: 4.5%, and the total extraction rate of PP I and PP II was 93.28%, RSD: 1.20%.	[153]
3	RE	PP VII/ PP H/ PP VI/ PP I/PP II	50 kg of heavy drug, plus 95% ethanol extraction 3 times, control the temperature between 70–80 °C, extraction time: 3–5 h, parallel extraction 3 times.	HPLC/orthogonal experimental method		The saponin content was PP VII: 90.86 mg/g, PP H: 198.02 mg/g, PP VI: 302.57 mg/g, PP I: 27.22 mg/g, PP II: 137.18 mg/g.	[154]
4	RE	PP I	Extraction medium: distilled water, 40~80% ethanol and anhydrous ethanol, solvent dosage: 8–20 times, extraction times: 1–3, extraction time: 1–3 h/time.	HPLC method	90% ethanol, 1:12 ratio, 1 h each time, 2 extractions. The order of effect size was as follows: number of extractions > extraction time > ratio > extraction solvent	PP I yield of 10.27%.	[155]
5	RE	PP I	Extraction medium: 55–95% ethanol solution, extraction temperature: 50–90 °C, extraction time: 1–3 h, herb particle size: powder, medium powder, coarse powder.	Colorimetric/Orthogonal experimental method	The ethanol concentration was 75%, the extraction temperature was 90 °C, the extraction time was 1.5 h, and the order of influence was as follows: extraction temperature > solvent concentration > extraction time	The total saponin yield was 12.74%, RSD: 1.90%.	[156]
6	RE	Rhizoma Paridis total saponins/ PP I/PP II	Extraction solvents: 30–100% methanol, soaking time before extraction: 30–150 min, liquid to material ratio: 10:1~60:1 (mL:g), extraction time: 30–90 min/time, extraction times: 1–4.	HPLC/orthogonal experimental/Response Surface method	The extraction solvent was 70% ethanol, the ratio of liquid to material was 30:1 (mL:g), and the extraction time was 60 min/times after continuous reflux extraction. The order of influence was as follows: extraction time > extraction times > extraction solvent > material to liquid ratio.	The extraction rate was 2.094%	[157]
7	RE	Rhizoma Paridis total saponins/ PP I/PP II/PP VI/PP VII	Extraction solvents: double-distilled water, 50~95% ethanol and anhydrous ethanol, Solvent dosage: 1:8–1:14 (g:mL), extraction time: 1–3 h, extraction times: 1–3 times.	HPLC/orthogonal experimental method	The extract was refluxed in a water bath with 75% ethanol for 1.5 h at a ratio of 1:12 (B:V) and extracted twice. The order of effect: Extraction times: > Extraction time > Material to liquid ratio > Extraction solvent	The total saponin yield was 10.33%, and PP I, II, VI, VII RSD were 1.3%, 0.70%, 0.94% and 0.58%.	[158]
8	USA-SE	Rhizoma Paridis total saponins	Material-liquid ratio: 1:30–1:110 (g:mL) add water-saturated *n*-butanol, placed in the ultrasonic cleaner, extraction temperature: 35–75 °C ultrasonic extraction for 30 min; ultrasonic power: 50–100%.	Colorimetric/L16(45) orthogonal experimental method	The crude powder (40 mesh) was soaked in 30% ethanol solution for 36 h and then sonicated for 30 min; the volume ratio of crude powder to (soaked) ethanol was 1:15. Influence size order was as follows: ultrasonic measurement > soaking time > extraction solvent > material to liquid ratio	The extraction rate of Rhizoma Paridis total saponins was 9.50%.	[159]
9	USA-SE	PP I/PP II/PP VI/PP VII	Methanol single material-liquid ratio: 1:5–1:15 (g:mL), single extraction time: 10~30 min, extraction temperature: 30–50 °C, extraction times: 1–~3.	HPLC/L9(34) orthogonal experimental method	The extraction was carried out for 20 min at an initial extraction temperature of 40 °C with a single material to liquid ratio of 15 times of methanol and the number of extractions was 3. The order of effect size was as follows: extraction number > initial extraction temperature > material to liquid ratio > single extraction time	The average recoveries of PP I, II/VI/VII were 101.04%, 101.73%, 98.69% and 101.17% with RSDs of 1.33%, 2.34%, 1.64%, and 2.17%, respectively.	[160]
10	USA-SE	Rhizoma Paridis total saponins	Solvent selection: 40–95% ethano, methanol, acetone, dosage selection: 1:10–1:30 (g:mL) of 75% ethanol, ultrasonic heating time: 20–60 min, ultrasonic temperature: 40~80 °C.	HPLC/Orthogonal experimental method	The extraction solvent was 75% ethanol, 15 times the amount of solvent, the extraction temperature was 70 °C, and the extraction time was 50 min. The order of effect size was as follows: extraction solvent > extraction time > extraction temperature > solvent times	The amount of extract was 0.2651 g, and the *Rhizoma Paridis* total saponins content was 0.2259%.	[161]
11	USA-SE	Rhizoma Paridis total saponins	Ethanol concentration: 60–80%, ethanol dosage: 1:30–1:70 (g:mL), extraction time: 15~60 min.	HPLC/L9 (34) Orthogonal experimental method	The extraction was performed by ultrasonication with 60% ethanol at a ratio of 1:50 material to liquid for 30 min. The order of effect size was as follows: solvent times > extraction solvent > extraction time	The total saponin yield was 4.387%.	[162]
12	Dip method	PP I/PP II	\	HPLC/Orthogonal experimental method	Take 50 g of the slices of Paris, macerate with 95% ethanol for 24 h with shaking, filter through, and fix the filtrate to 500 mL with 95% ethanol.	The total amount of PP I/PP IIwas 0.356%.	[163]
13	USA-SE		\	HPLC/Orthogonal experimental method	Take 50 g of the slices of Paris, sonicate it with 95% ethanol at 500 mL (power 250 W, frequency 33 kHz) for 30 min, let it cool, filter it, and fix the filtrate to 500 mL with 95% ethanol.	The total amount of PP I/PP II was 1.287%.	
14	RE		\	HPLC/Orthogonal experimental method	Take 50 g of the slices of Paris, add 250 mL of 95% ethanol, and reflux twice, 2 h each time. Combine the extracts, filter, and dilute the filtrate to 500 mL with 95% ethanol. There was a significant difference in the ethanol volume fraction factor	The total amount of PP I/PP II was 1.192%, RSD was 2.24%.	
15	MWA-SE	Rhizoma Paridis total saponins	Solvent selection: 45–90% ethanol, extraction temperature: 60–80 °C, solvent dosage: 1:10–1:20 (g:mL).	Colorimetric/L9(34) Orthogonal experimental method	The extraction was carried out at 80 °C for 20 min with 10 times the amount of 60% ethanol solution. The order of effect size was as follows: ethanol concentration > extraction temperature > extraction time > ethanol dosage.	The total saponin yield was 11.6% and the RSD was 0.3%	[164]
16	MWA-SE	PP I/PP VII	Extraction solvent: 0~100% ethanol concentration, microwave power: 300~500 w, liquid-solid ratio: 1:10–1:30 (g:mL), radiation time: 5–15 min, extraction temperature: 30–50 °C.	HPLC/LC-ESI-MS/H NMR Spectroscopic	70% *v*/*v* ethanol aqueous solution concentration, 300 W microwave power, 20:1 mL/g liquid–solid ratio, 50 °C extraction temperature, and 15 min irradiation time. The extraction solvent was the decisive factor, while microwave power and irradiation time were important factors in MAE.	The extraction yields of PP VII and PP I were 5.66 and 15.4 mg/g.	[165]
17	Room-temperature UPE	Saponin A, saponin B, saponin C, saponin D	Extraction solvent: water, methanol, 95% ethanol, water-saturated *n*-butanol, ethanol concentration: 30–95%, UHP pressure: 100–600 Mpa, extraction time: 1–5 min.	HPLC/L9 (34) Orthogonal experimental method	The ethanol concentration was 90%, the extraction pressure was 400 Mpa, the extraction time was 2 min, and the liquid–solid ratio was 40:1.	The rates of saponin A, saponin B, saponin C, and saponin D were 1.164%, 0.591%, 0.043%, and 0.053% respectively.	[166]
18	UHPA-SE		\	HPLC method	Treat with 40 mL of 90% ethanol in water (*v*/*v*), fix the transducer, and sonicate the system in an ultrasonic bath (frequency, 50 Hz; power, 250 W) for 30 min.	The rates of saponin A, saponin B, saponin C, and Saponin D were 1.113%, 0.513%, 0.038%, and 0.045% respectively.	
19	Microwave-assisted extraction (MAE).		\	HPLC method	Using a 40 mL 90% ethanol aqueous solution (*v*/*v*) treatment, the system was exposed to a microwave radiation source operating at 300 W and 2450 MHz frequency and subjected to atmospheric pretreatment for 15 min of extraction.	The rates of saponin A, saponin B, saponin C, and saponin D were 1.139%, 0.539%, 0.041%, and 0.049%, respectively.	
20	Method of Sohxlet extraction.		\	HPLC method	Boil the mixture for 3 h with 80 mL of 90% ethanol in water (*v*/*v*),	The rates of saponin A, saponin B, saponin C, and saponin D were 1.119%, 0.511%, 0.039%, and 0.044%, respectively.	
21	Usual room-temperature extraction (RTE)		\	HPLC method	Extraction with 40 mL of 90% ethanol in water (*v*/*v*), 25 °C room-temperature for 5 days.	The rates of saponin A, saponin B, saponin C, and saponin D were 0.988%, 0.483%, 0.034%, and 0.040%, respectively.	
22	SFE-CO_2_	Pennogenin	Extraction method: dynamic, static, dynamic + static, entrainment agent: methanol, acetone, ethanol, ethyl acetate, extraction temperature: 5–65 °C, extraction pressure: 150–400 bar, extractant dosage: 100–600 mL, extraction time: 2–7 h, SC-CO_2_ flow rate: 15–60 g/min.	HPLC/L9 (34) Orthogonal experimental method	The extraction was carried out by a combination of dynamic and static methods with the optimal ratio of ethanol 90%–ethyl acetate 10%, an extraction temperature of 52 °C, an extraction pressure of 315 bar, a SC-CO_2_ flow rate of 39 g/min, an extractant dosage of 300 mL and an extraction time of 4 h.	The yield of Pennogenin was 16.32%.	[167]
23	AEE (ultrasound-assisted extraction)	Rhizoma Paridis total saponins	Enzyme dosage:10–50 U/g substrate, enzyme digestion temperature: 30–70 °C, PH: 3.5–5.5, enzyme digestion time: 30–150 min.	Colorimetric/L9(34) Orthogonal experimental/Response Surface method	The amount of cellulase was 32 U/g of substrate, the enzymatic digestion temperature was 52 °C, the enzymatic pH was 4.6, and the enzymatic digestion time was 92 min.	The theoretical value of the extraction rate of Rhizoma Paridis total saponins was 1.66%.	[168]
24	AEE (water extraction)	PP I	Extraction time: 1–3 h, extraction times: 1–3, extraction temperature: 60–95 °C, solvent dosage: 1:5–1:8 (g:mL), PH value: 5–9, drug particle size: 10–40.	Colorimetric/L9(34) Orthogonal experimental method	Enzyme A and enzyme B were used alternately, where the dosage of enzyme A was 1 mL/kg of raw drug and the dosage of enzyme B was 2 mL/kg of raw drug, crushed into 20 items of herb with 7 times the amount of solvent pH 7 extracted 3 times at 60 °C for 3 h each.	The extraction rate was increased by 24.28% to 61.27%.	[169]

Note: Reflux extraction (RE), ultrasonic assisted solvent extraction (USA-SE), microwave assisted solvent extraction (MWA-SE), ultrahigh pressure assisted solvent extraction (UHPA-SE), supercritical fluid CO_2_ extraction (SFE-CO_2_), aqueous enzymatic extraction (AEE).

## 7. Conclusions and Future Perspectives

A valuable Chinensis herb, Paris is a perennial herb that comes in two types: wild and cultivated. Paris contains various active ingredients, with saponins being the main active ingredients. However, due to the slow growth of wild Paris and excessive consumption, Paris is a scarce resource. Therefore, improving saponins’ purity and extraction rate is an important aspect of Paris research.

*P. polyphylla* saponins are mainly used in anti-cancer, anti-fibrotic, and antibacterial treatments, but their applications in acne are not yet mature. Nevertheless, the overview of the pathogenesis of acne suggests that such ingredients in *P. polyphylla* could be used as potential agents in treating acne.

In addition, *P. polyphylla* saponins are diverse and have different biological activities due to their different structures. The main chemical components related to the pathogenesis of acne are PP I, PP II, PP VI, PP VII, diosgenin, and so on. Based on in vitro and in vivo studies, *P. polyphylla* saponins inhibited inflammatory responses caused by different signaling pathways (NF-κB, PI3K/Akt, MAPK) and showed strong antibacterial effects against *P. acnes*, *S. aureus*, and *S. epidermidis*. It also modulates local T-cell factor disorders caused by immune dysregulation and has an immunomodulatory effect on the immune response to cells caused by Th1/Th17 imbalance. It also modulates the production of free radicals and reactive oxygen species caused by lipid peroxidation. This overview reveals that *P. polyphylla* saponins have a tremendous potential in the pathogenesis of anti-acne treatment. At the same time, we hope it can provide a quick reference for the experimental design of future studies.

Although it has been suggested that *P. polyphylla* saponins may have an anti-inflammatory, antibacterial, anti-hyperkeratosis, immunomodulatory, and antioxidant effect on acne pathogenesis, previous studies have not demonstrated the effects of *P. polyphylla* saponins on skin problems caused by sebum hyperproduction. This leaves the mechanism of *P. polyphylla* saponins in seborrheic acne unclear.

In order to establish the relationship between *P. polyphylla* saponins and acne pathogenesis, studies with direct relevance to acne should be designed, leading to further studies on the chemical composition and biological activities of *P. polyphylla* saponins.

## Data Availability

The data presented in this study are available on request from the corresponding author.

## Supplementary Materials

The following supporting information can be downloaded at: https://www.mdpi.com/article/10.3390/molecules29081793/s1, S1. Organic Solvent Extraction Methods; S2. Physical Extraction Methods; S3. Aqueous Enzymatic Extraction (AEE). References [170,171,172,173,174,175,176,177,178,179,180,181,182] are cited in Supplementary Materials.

## References

[1] Oulès B., Philippeos C., Segal J., Tihy M., Vietri Rudan M., Cujba A., Grange P.A., Quist S., Natsuga K., Deschamps L. (2020). Contribution of GATA6 to homeostasis of the human upper pilosebaceous unit and acne pathogenesis. Nat. Commun..

[2] Min K.J., Ha S., Son J.A., Ji H.S., Houh Y., Cho E., Ji H.C., Yoon S.R., Yang Y., Bang S.I. (2012). Polyphenon-60 displays a therapeutic effect on acne by suppression of TLR2 and IL-8 expression via down-regulating the ERK1/2 pathway. Arch. Dermatol. Res..

[3] Ruan S.F., Xiang S.J., Wu W.F., Cao S.W., Liu Q. (2020). Potential role of mTORC1 and the PI3K-Akt pathway in anti-acne properties of licorice flavonoids. J. Funct. Foods.

[4] Gabsik Y., Hye E.L., Sang H.Y., Han C. (2018). Licochalcone A attenuates acne symptoms mediated by suppression of NLRP3 inflammasome. Phytother. Res..

[5] Weber N., Biehler K., Schwabe K., Haarhaus B., Quirin K.W., Frank U., Schempp C., Wölfle U. (2019). Hop Extract Acts as an Antioxidant with Antimicrobial Effects against *Propionibacterium acnes* and *Staphylococcus aureus*. Molecules.

[6] Jeong W.Y., Kim K. (2017). Anti-Propionibacterium acnes and the anti-inflammatory effect of *Aloe ferox* miller components. J. Herb. Med..

[7] Kwon H.C., Kim T.Y., Lee C.M., Lee K.S., Lee K.K. (2019). Active compound chrysophanol of Cassia tora seeds suppresses heat-induced lipogenesis via inactivation of JNK/p38 MAPK signaling in human sebocytes. Lipids Health Dis..

[8] Man S.L., Chai H.Y., Cui J.X., Yao J.W., Ma L., Gao W.Y. (2018). Antitumor and anti-metastatic mechanisms of *Rhizoma paridis* saponins in *Lewis mice*. Environ. Toxicol..

[9] Qin X.J., Sun D.J., Ni W., Chen C.X., Hua Y., He L., Liu H.Y. (2012). Steroidal saponins with antimicrobial activity from stems and leaves of *Paris polyphylla* var. yunnanensis. Steroids.

[10] Yang L., Shou Y., Yang Y., Xu J. (2021). Elucidating the immune infiltration in acne and its comparison with rosacea by integrated bioinformatics analysis. PLoS ONE.

[11] Lan M. (2017). Dian Nan Ben Cao.

[12] Liu X., Li Z. (2017). Agriculture God’s Canon of Materal Medica.

[13] Li S. (2013). Compendium of Materal Medica.

[14] China Pharmacopoeia Commission (2015). Pharmacopoeia of the People’s Republic of China.

[15] Puwein A., Thomas S.C. (2020). An Overview of Paris polyphylla, a Highly Vulnerable Medicinal Herb of Eastern Himalayan Region for Sustainable Exploitation. J. Nat. Prod..

[16] He L., Shi W., Liu X., Zhao X., Zhang Z. (2018). Anticancer action and mechanism of ergosterol peroxide from *Paecilomyces cicadae* fermentation broth. Int. J. Mol. Sci..

[17] Malla B., Gauchan D.P., Chhetri R.B. (2015). An ethnobotanical study of medicinal plants used by ethnic people in Parbat district of western Nepal. J. Ethnopharmacol..

[18] Gao W. (2014). The clinic study of Chong, Lou, Ji, Cai, Sheng, Hua, Tang in promoting uterine recovery after abortion surgery. Contemp. Med..

[19] Guo T., Zhou Z., Huang Z., Wen H., Huang X. (2007). Effect of Lianxiuwugong Dilong Tang on the immune function of children with Mycoplasma pneumonia. New Chin. Med..

[20] Su A., Duan H. (2000). Qingwenjiedutonglin soup treated with 196 cases of gonorrhea. Mod. Chin. Med..

[21] Zhou L., Song H., Zhang Y., Ren Z., Li M., Fu Q. (2020). Polyphyllin VII attenuated RANKL-induced osteoclast differentiation via inhibiting of TRAF6/c-Src/PI3K pathway and ROS production. BMC Musculoskel. Dis..

[22] Wang Y., Yi T., Lin K. (2011). In Vitro Activity of *Paris polyphylla* Smith Against Enterovirus 71 and Coxsackievirus B3 and Its Immune Modulation. Am. J. Chin Med..

[23] Sun D., Tu Y., He L. (2013). Antimicrobial activity of ethanol extract from Dianchonglou on acne-causing bacteria. Dermatol. Vener..

[24] Deng D., Lauren D.R., Cooney J.M., Jensen D.J., Wurms K.V., Upritchard J.E., Cannon R.D., Wang M.Z., Li M.Z. (2008). Antifungal Saponins from *Paris polyphylla* Smith. Planta Med..

[25] Sun C., Ni W., Yan H., Liu Z., Yang L., Si Y., Hua Y., Chen C., He L., Zhao J. (2014). Steroidal saponins with induced platelet aggregation activity from the aerial parts of *Paris verticillata*. Steroids.

[26] Zhang C., Li C.Y., Jia X.J., Wang K., Tu Y.B., Wang R.C., Liu K.C., Lu T., He C.W. (2019). In Vitro and In Vivo Anti-Inflammatory Effects of Polyphyllin VII through Downregulating MAPK and NF-κB Pathways. Molecules.

[27] He H., Xu C., Zheng L., Wang K., Jin M., Sun Y., Yue Z. (2020). Polyphyllin VII induces apoptotic cell death via inhibition of the PI3K/Akt and NF-κB pathways in A549 human lung cancer cells. Mol. Med. Rep..

[28] Yang S., Jiang Y., Yu X., Zhu L., Wang L., Mao J., Wang M., Zhou N., Yang Z., Liu Y. (2021). Polyphyllin I Inhibits Propionibacterium acnes-Induced IL-8 Secretion in HaCaT Cells by Downregulating the CD36/NOX1/ROS/NLRP3/IL-1β Pathway. Evid.-Based Complement. Altern. Med..

[29] Ichiro K., Layton L.M., Rei O. (2021). Updated Treatment for Acne: Targeted Therapy Based on Pathogenesis. Dermatol. Ther..

[30] Unkles S.E., Gemmell C.G. (1982). Effect of clindamycin, erythromycin, lincomycin, and tetracycline on growth and extracellular lipase production by propionibacteria in vitro. Antimicrob. Agents Chemother..

[31] Batra R., Sadhasivam S., Saini S., Gupta S., Bisen R., Sinha M., Ghosh S., Jain S. (2020). Efficacy and Safety of VB-1953 Topical Gel in Non-Responder Acne Patients with Clindamycin-Resistant *Cutibacterium acnes*. Drugs R D.

[32] Hernandez Ceruelos A., Romero-Quezada L.C., Ruvalcaba Ledezma J.C., Lopez Contreras L. (2019). Therapeutic uses of metronidazole and its side effects: An update. Eur. Rev. Med. Pharmacol..

[33] Lowe L., Herbert A.A. (1989). Cystic and comedonal acne: A side effect of etretinate therapy. Int. J. Dermatol..

[34] Plewig G., Schopf E. (1975). Anti-inflammatory effects of antimicrobial agents: An in vivo study. J. Investig. Dermatol..

[35] Shen S., Chen D., Li X., Li T., Yuan M., Zhou Y., Ding C. (2014). Optimization of extraction process and antioxidant activity of polysaccharides from leaves of *Paris polyphylla*. Carbohyd. Polym..

[36] Zhang Z., Chu J., Zhou Y., Di W., Li X., Peng X., Li C., Ju R. (2022). Study on Extraction Technology and Antioxidant Effect of Flavonoids from Chonglou (*Petiolate paris*). Guid. J. Tradit. Chin Med. Phar..

[37] Zhu T., Wu W., Yang S., Li D., Sun D., He L. (2019). Polyphyllin I Inhibits *Propionibacterium acnes*-Induced Inflammation In Vitro. Inflammation.

[38] Das Gupta D., Mishra S., Verma S.S., Shekher A., Rai V., Awasthee N., Das T.J., Paul D., Das S.K., Tag H. (2021). Evaluation of antioxidant, anti-inflammatory and anticancer activities of diosgenin enriched *Paris polyphylla* rhizome extract of Indian Himalayan landraces. J. Ethnopharmacol..

[39] Ding Y.G., Zhao Y.L., Zhang J., Zuo Z.T., Zhang Q.Z., Wang Y.Z. (2021). The traditional uses, phytochem, and pharmacological properties of *Paris* L. (Liliaceae): A review. J. Ethnopharmacol..

[40] Zhai X., Wang K., Gao X., Yan B. (2023). Research Progress on Chemical Constituents and Pharmacological Activities of *Menispermi rhizoma*. Molecules.

[41] Yue H., Ding C., Yang R., Zhang L., Zhou Y., Li Y. (2011). Karyomorphology of some taxa of *Paris* (Melanthiaceae) from Sichuan province, China. Caryologia.

[42] Shah A.S., Mazumder P.B., Choudhury M.D. (2012). Medicinal properties of *Paris polyphylla* smith: A review. J. Herb. Med. Toxicol..

[43] Mohd T., Belwal T., Bhatt I.D., Pande V., Nandi S.K. (2018). Polyphenolics in leaves of Paris polyphylla: An important high value Himalayan medicinal herb. Ind. Crop. Prod..

[44] Tariq M., Nandi S.K., Bhatt I.D., Bhavsar D., Roy A., Pande V. (2021). Phytosociological and niche distribution study of *Paris polyphylla* smith, an important medicinal herb of Indian Himalayan region. Trop. Ecol..

[45] Wang Y., Zhang J., Shen T., Zhang J. (2022). Biomass allocation and allometry of *Paris polyphylla* var. yunnanensis with different ages. Chin. Tradit. Herb. Drugs.

[46] Wu Z., Zhang J., Xu F., Wang Y., Zhang J. (2017). Rapid and simple determination of polyphyllin I, II, VI, and VII in different harvest times of cultivated *Paris polyphylla* Smith var. *yunnanensis* (Franch.) Hand.-Mazz by UPLC-MS/MS and FT-IR. J. Nat. Med..

[47] Dreno B., Dagnelie M.A., Khammari A., Corvec S. (2020). The Skin Microbiome: A New Actor in Inflammatory Acne. Am. J. Clin. Dermatol..

[48] Huang T.Y., Jiang Y.E., Scott D.A. (2022). Culturable bacteria in the entire acne lesion and short-chain fatty acid metabolites of *Cutibacterium acnes* and *Staphylococcus epidermidis* isolates. Biochem. Biophys. Res. Commun..

[49] Gannesen A.V., Zdorovenko E.L., Botchkova E.A., Hardouin J., Massier S., Kopitsyn D.S., Gorbachevskii M.V., Kadykova A.A., Shashkov A.S., Zhurina M.V. (2019). Composition of the Biofilm Matrix of *Cutibacterium acnes* Acneic Strain RT5. Front. Microbiol..

[50] Akaza N., Akamatsu H., Numata S., Matsusue M., Mashima Y., Miyawaki M., Yamada S., Yagami A., Nakata S., Matsunaga K. (2014). Fatty acid compositions of triglycerides and free fatty acids in sebum depend on amount of triglycerides, and do not differ in presence or absence of acne vulgaris. J. Dermatol..

[51] Li Y.H., Sun Y., Tang T.L., Niu Y.B., Li X.Q., Xie M., Jin H.C., Mei Q.B. (2019). Paris saponin VII reverses chemoresistance in breast MCF-7/ADR cells. J. Ethnopharmacol..

[52] Xia P.P., Wu Y.P., Lian S.Q., Yan L., Meng X., Duan Q.D., Zhu G.Q. (2021). Research progress on Toll-like receptor signal transduction and its roles in antimicrobial immune responses. Appl. Microbiol. Biotechnol..

[53] Zhang P., The E., Nedumaran B., Ao L., Jarrett M.J., Xu D., Fullerton D.A., Meng X. (2020). Monocytes enhance the inflammatory response to TLR2 stimulation in aortic valve interstitial cells through paracrine up-regulation of TLR2 level. Int. J. Biol. Sci..

[54] Jang M., Hwang I., Hwang B., Kim G. (2020). Anti-inflammatory effect of *Antirrhinum majus* extract in lipopolysaccharide-stimulated RAW 264.7 macrophages. Food Sci. Nutr..

[55] Lee J.W., Kang Y.J., Choi H.K., Yoon Y.G. (2018). Fractionated Coptis chinensis Extract and Its Bioactive Component Suppress Propionibacterium acnes-Stimulated Inflammation in Human Keratinocytes. J. Microbiol. Biotechnol..

[56] Jin S., Lee M.Y. (2018). The ameliorative effect of hemp seed hexane extracts on the *Propionibacterium acnes*-induced inflammation and lipogenesis in sebocytes. PLoS ONE.

[57] Tsai H.H., Lee W.R., Wang P.H., Cheng K.T., Chen Y.C., Shen S.C. (2013). Propionibacterium acnes-induced iNOS and COX-2 protein expression via ROS-dependent NF-κB and AP-1 activation in macrophages. J. Dermatol. Sci..

[58] Jeremy A., Holland D.B., Roberts S.G., Thomson K.F., Cunliffe W.J. (2003). Inflammatory events are involved in acne lesion initiation. J. Investig. Dermatol..

[59] Zouboulis C.C., Coenye T., He L., Kabashima K., Kobayashi T., Niemann C., Nomura T., Olah A., Picardo M., Quist S.R. (2022). Sebaceous immunobiology-skin homeostasis, pathophysiology, coordination of innate immunity and inflammatory response and disease associations. Fron. Immunol..

[60] Kistowska M., Meier B., Proust T., Feldmeyer L., Cozzio A., Kuendig T., Contassot E., French L.E. (2015). Propionibacterium acnes Promotes Th17 and Th17/Th1 Responses in Acne Patients. J. Investig. Dermatol..

[61] Agak G.W., Qin M., Nobe J., Kim M., Krutzik S.R., Tristan G.R., Elashoff D., Garbán H.J., Kim J. (2014). Propionibacterium acnes Induces an IL-17 Response in Acne Vulgaris that Is Regulated by Vitamin A and Vitamin D. J. Investig. Dermatol..

[62] Karadag A.S., Ertugrul D.T., Bilgili S.G., Takci Z., Akin K.O., Calka O. (2012). Immunoregulatory effects of isotretinoin in patients with acne. Br. J. Dermatol..

[63] Bohm M., Luger T.A. (1998). The pilosebaceous unit is part of the skin immune system. Dermatology.

[64] Lawrence D., Shaw M., Katz M. (1986). Elevated free testosterone concentration in men and women with acne vulgaris. Clin. Exp. Dermatol..

[65] Ceruti J.M., Leirós G.J., Balañá M.E. (2018). Androgens and androgen receptor action in skin and hair follicles. Mol. Cell. Endocrinol..

[66] Kamei Y., Miura S., Suganami T., Akaike F., Kanai S., Sugita S., Katsumata A., Aburatani H., Unterman T.G., Ezaki O. (2008). Regulation of SREBP1c gene expression in skeletal muscle: Role of retinoid X receptor/liver X receptor and forkhead-O1 transcription factor. Endocrinology.

[67] Juhl C.R., Bergholdt H., Miller I.M., Jemec G., Kanters J.K., Ellervik C. (2018). Dairy Intake and Acne Vulgaris: A Systematic Review and Meta-Analysis of 78,529 Children, Adolescents, and Young Adults. Nutrients.

[68] Hu X., Weng X., Tian Y., Wang C., Yang Y., Xu K., Zhang C. (2019). Effects of omega-3 polyunsaturated fatty acids on steroidogenesis and cellular development in PCOS rats. Food Funct..

[69] Mirdamadi Y., Thielitz A., Wiede A., Goihl A., Papakonstantinou E., Hartig R., Zouboulis C.C., Reinhold D., Simeoni L., Bommhardt U. (2015). Insulin and insulin-like growth factor-1 can modulate the phosphoinositide-3-kinase/Akt/FoxO1 pathway in SZ95 sebocytes in vitro. Mol. Cell. Endocrinol..

[70] Firlej E., Kowalska W., Szymaszek K., Roliński J., Bartosińska J. (2022). The Role of Skin Immune System in Acne. J. Clin. Med..

[71] Kretzschmar K., Cottle D.L., Schweiger P.J., Watt F.M. (2015). The Androgen Receptor Antagonizes Wnt/β-Catenin Signaling in Epidermal Stem Cells. J. Investig. Dermatol..

[72] Wang W., Liu Y., You L., Sun M., Qu C., Dong X., Yin X., Ni J. (2020). Inhibitory effects of Paris saponin I, II, VI and VII on HUVEC cells through regulation of VEGFR2, PI3K/AKT/mTOR, Src/eNOS, PLC gamma/ERK/MERK, and JAK2-STAT3 pathways. Biomed. Pharmacother..

[73] Chang J., Li Y., Wang X., Hu S., Wang H., Shi Q., Wang Y., Yang Y. (2017). Polyphyllin I suppresses human osteosarcoma growth by inactivation of Wnt/beta-catenin pathway in vitro and in vivo. Sci. Rep..

[74] Laly A.C., Sliogeryte K., Pundel O.J., Ross R., Keeling M.C., Avisetti D., Waseem A., Gavara N., Connelly J.T. (2021). The keratin network of intermediate filaments regulates keratinocyte rigidity sensing and nuclear mechanotransduction. Sci. Adv..

[75] Maruthappu T., Chikh A., Fell B., Delaney P.J., Brooke M.A., Levet C., Moncada-Pazos A., Ishida-Yamamoto A., Blaydon D., Waseem A. (2017). Rhomboid family member 2 regulates cytoskeletal stress-associated Keratin 16. Nat. Commun..

[76] Hughes B.R., Morris C., Cunliffe W.J., Leigh I.M. (1996). Keratin expression in pilosebaceous epithelia in truncal skin of acne patients. Br. J. Dermatol..

[77] Caillon F., O’Connell M., Eady E.A., Jenkins G.R., Cove J.H., Layton A.M., Mountford A.P. (2010). Interleukin-10 secretion from CD14+ peripheral blood mononuclear cells is downregulated in patients with acne vulgaris. Br. J. Dermatol..

[78] Akaza N., Akamatsu H., Kishi M., Mizutani H., Ishii I., Nakata S., Matsunaga K. (2009). Effects of Propionibacterium acnes on various mRNA expression levels in normal human epidermal keratinocytes in vitro. J. Dermatol..

[79] Komine M., Rao L.S., Freedberg I.M., Simon M., Milisavljevic V., Blumenberg M. (2001). Interleukin-1 induces transcription of keratin K6 in human epidermal keratinocytes. J. Investig. Dermatol..

[80] Li H. (1998). Chonglou. Genus. Plants.

[81] Huang W., Zhou J. (1962). Study on the steroidal saponin ligand composition of Shigarou. Med. Phar. Yunnan.

[82] Huang X., Gao W., Man S., Zhao Z. (2009). Advances in studies on saponins in plants of Paris Land their biosynthetic approach. Chin. Tradit. Herb. Drugs.

[83] Chen C., Zhou J., Zhang Y., Gao C. (1983). Studies on the saponin composition of plants in yunnan vim steroidal saponins in paris luquanensis. Plant Divers..

[84] Wang Y., Gao W., Yuan L., Liu X., Wang S., Chen C. (2007). Chemical constituents from rhizome of *Paris polyphylla* var. yunnanensis. Chin. Tradit. Herb. Drugs.

[85] Huang X., Gao W., Man S., Yan J., Wang Y. (2009). Chemical constituents from herbs of *Paris verticillata*. China J. Chin. Mater. Med..

[86] Sha A., Liu Y., Qiu X., Xiong B. (2023). Polysaccharide from *Paris polyphylla* improves learning and memory ability in D-galactose-induced aging model mice based on antioxidation, p19/p53/p21, and Wnt/β-catenin signaling pathways. Int. J. Biol. Macromol..

[87] Wu X., Wang L., Wang G., Wang H., Dai Y., Yang X., Ye W., Li Y. (2013). Triterpenoid saponins from rhizomes of *Paris polyphylla* var. yunnanensis. Carbohydr. Res..

[88] Thuy T.T.V., Lien T.K.V., Quan H.N., Khang V.P., Dung T.N., Lan T.N.N., Mau H.C. (2019). Cytotoxic effects of steroidal glycosides isolated from the Paris vietnamensis plant on cancer cell lines and against bacterial strains. Biotechnol. Biotechnol. Equip..

[89] Huang X., Gao W., Man S., Gao Y., Huang L., Liu C. (2011). Isolation and Identification of Compounds Present in Rhizomes of *Paris axialis* H. Li and Study of Their Cytotoxic Effects. Lat. Am. J. Pharm..

[90] Yang Y., Zhao Y., Zuo Z., Wang Y. (2019). Determination of Total Flavonoids for *Paris polyphylla* var. Yunnanensis in Different Geographical Origins Using UV and FT-IR Spectroscopy. J. AOAC Int..

[91] Liu Y., Qiu P., Wang M., Lu Y., He H., Tang H., Zhang B. (2021). New Steroidal Saponins Isolated from the Rhizomes of *Paris mairei*. Molecules.

[92] Zhou L.A., Yang C.Z., Li J.Q., Wang S.L., Wu J.Y. (2003). Heptasaccharide and octasaccharide isolated from *Paris polyphylla* var. yunnanensis and their plant growth-regulatory activity. Plant Sci..

[93] Yan L., Gao W., Zhang Y., Wang Y. (2008). A new phenylpropanoid glycosides from *Paris polyphylla* var. yunnanensis. Fitoterapia.

[94] Qin X., Ni W., Chen C., Liu H. (2018). Seeing the light: Shifting from wild rhizomes to extraction of active ingredients from above-ground parts of *Paris polyphylla* var. yunnanensis. J. Ethnopharmacol..

[95] Song W., Zheng W., Zhang J., Zhang T., Liu S., Yu L., Ma B. (2018). Metabolism of saponins from traditional Chin medicines: A review. Acta Pharm. Sin..

[96] Osbourn A. (1996). Saponins and plant defence—A soap story. Trends Plant Sci..

[97] Tian W.S., Xu Q.H., Chen L., Zhao C.F. (2004). Synthesis of pennogenin utilizing the intact skeleton of diosgenin. Sci. China Ser. B Chem..

[98] Liu F., Li L., Tian X., Zhang D., Sun W., Jiang S. (2021). Chemical Constituents and Pharmacological Activities of Steroid Saponins Isolated from. J. Chem..

[99] Singh S.B., Thakur R.S., Schulten H. (1980). Spirostanol saponins from *Paris polyphylla*, structures of polyphyllin C, D, E and F. Phytochem.

[100] Viet Cuong L.C., Nhi N.P.K., Ha T.P., Anh L.T., Dat T.T.H., Oanh P.T.T., Phuong N.T.M., Thu V.T.K., Duc H.V., Anh H.L.T. (2022). A new steroidal saponin from the aerial parts of *Solanum torvum*. Nat. Prod. Res..

[101] Yu L., Li Y., Gao W., Ling S., Ni W., Ji Y., Liu H. (2022). Steroidal saponins with cytotoxic activity from the stems and leaves of *Paris fargesii*. New J. Chem..

[102] Singh S.B., Thakur R.S., Schulten H.R. (1982). Furostanol saponins from *Paris polyphylla*: Structures of polyphyllin G and H. Phytochem.

[103] Xu X., Li T., Fong C.M.V., Chen X., Chen X., Wang Y., Huang M., Lu J. (2016). Saponins from Chin Medicines as Anticancer Agents. Molecules.

[104] Jing S., Wang Y., Li X., Man S., Gao W. (2017). Chemical constituents and antitumor activity from *Paris polyphylla* Smith var. yunnanensis. Nat. Prod. Res..

[105] Tuchayi S.M., Makrantonaki E., Ganceviciene R., Dessinioti C., Feldman S.R., Zouboulis C.C. (2015). Acne vulgaris. Nat. Rev. Dis. Prim..

[106] Williams H.C., Dellavalle R.P., Garner S. (2012). Acne vulgaris. Lancet.

[107] Manhong Z., Du W., Long S., Han J., Shen Y., Li T. (2008). Effect of Rhizoma Paridis Total Saponins on TNF-α and IL-1β Secretion in Rat Peritoneal Macrophages Induced by Lipopolysaccharide. J. Sichuan Tradit. Chin. Med..

[108] Leisheng W., Tian M., Yanpin Z., Shiqiao L., Yu L., Shaoxian L. (2015). Diosgenin inhibits IL-1β-induced expression of inflammatory mediators in human osteoarthritis chondrocytes. Int. J. Clin. Exp. Pathol..

[109] Long J., Pi X., Tu Y. (2016). Effect of Polyphylin I on proliferation and expressions of HIF-1α, VEGF in laryngeal carcinoma cell line Hep-2 under hypoxia. Acta Univ. Med. Anhui.

[110] Wu L., Peng X., Ju R., Yang S., Li H. (2022). Optimization of microwave ultrasound-assisted extraction of paris and its anti-inflammatory activity by response surface methodology. J. Chin Med. Mater..

[111] Li Y., Sun Y., Fan L., Chen X., Mei Q., Yue Z., Li Z., Zheng H., Tang Y. (2015). Effects of Polyphyllin VI on Metastasis of colon Cancer Cells LoVo and the Possible Mechanisms. Milit. Med. J. South China.

[112] Zhang C., Li Q.R., Qin G.Z., Zhang Y., Li C.Y., Han L.W., Wang R.C., Wang S.D., Chen H.X., Liu K.C. (2021). Anti-angiogenesis and anti-metastasis effects of Polyphyllin VII on *Hepatocellular carcinoma* cells in vitro and in vivo. Chin. Med..

[113] Choi K.W., Park H.J., Jung D.H., Kim T.W., Park Y.M., Kim B.O., Sohn E.H., Moon E.Y., Um S.H., Rhee D.K. (2010). Inhibition of TNF-alpha-induced adhesion molecule expression by diosgenin in mouse vascular smooth muscle cells via downregulation of the MAPK, Akt and NF-κB signaling pathways. Vasc. Pharmacol..

[114] Liu W.L., Zhu M., Yu Z.W., Yin D., Lu F.F., Pu Y.Y., Zhao C., He C., Cao L. (2017). Therapeutic effects of diosgenin in experimental autoimmune encephalomyelitis. J. Neuroimmunol..

[115] Quan Q., Weng D., Li X., An Q., Yang Y., Yu B., Ma Y., Wang J. (2022). Analysis of drug efficacy for inflammatory skin on an organ-chip system. Front. Bioeng. Biotechnol..

[116] Yang Y., Wang C., Wang J., Yang L., Lv Z., An Q., Wang Y., Shao X., Wang F., Huo T. (2024). Rhizoma Paridis saponins attenuate Gram-negative bacteria-induced inflammatory acne by binding to KEAP1 and modulating Nrf2 and MAPK pathways. J. Cell. Mol. Med..

[117] Kuehnast T., Cakar F., Weinhäupl T., Pilz A., Selak S., Schmidt M.A., Rüter C., Schild S. (2018). Comparative analyses of biofilm formation among different *Cutibacterium acnes* isolates. Int. J. Med. Microbiol..

[118] Cai X.Q., Guo L.L., Pei F., Chang X.Y., Zhang R. (2018). Polyphyllin G exhibits antimicrobial activity and exerts anticancer effects on human oral cancer OECM-1 cells by triggering G2/M cell cycle arrest by inactivating cdc25C-cdc2. Arch. Biochem. Biophys..

[119] Wang Y., Fan Q., Liu B., Hu J., Zhang C. (2022). Antibacterial Effects of Different Saponins from *Paris polyphylla* on Acne-associated Pathogens In Vitro. Chin. Dermat. Int. Tradit. West. Med..

[120] Wang Q., Sun D., He L., Xu D., Wu W., Li Q., Liu H., Du Y. (2016). Evaluation of the Inhibitory Effect of Total Saponins of Polyphylla and its Fraction on Acne-associated Pathogens. Chin. J. Dermatovenereol..

[121] Carlavan I., Bertino B., Rivier M., Martel P., Bourdes V., Motte M., Déret S., Reiniche P., Menigot C., Khammari A. (2018). Atrophic scar formation in patients with acne involves long-acting immune responses with plasma cells and alteration of sebaceous glands. Br. J. Dermatol..

[122] Wang J., Liu R., Xiao H., Bi H., Li Z. (2010). The effects of Paridis saponin II on the production of cell cytokines in CD4^+^CD25^+^ T regulation cells from lupus nephritis patients’ peripheral blood. Prog. Mod. Biomed..

[123] Li C. (2011). The Study on Anti-Breast Cancer and Immunomodulating Effects of Rhizom Paridis Saponins. Ph.D. Thesis.

[124] Fallon M.E., Hinds M.T. (2021). Single cell morphological metrics and cytoskeletal alignment regulate VCAM-1 protein expression. Biochem. Biophys. Res. Commun..

[125] Hart J. (1999). Comparative study of serum soluble VCAM-1 and ICAM-1 levels in the early neonatal period. Acta Paediatr..

[126] Wu B., Yang X., Cao Y., Yu T., Xi A., Cheng L., Jin Y., Chen Q. (2012). Clinical and Immunohistochemical Research of Piyan Xiaojin Decoction-2 Combining with Narrow-Spectrum UVB Exposure Treating Atopic Dermatitis (AD) with Blood Deficiency and Wind Dryness. Chin. Arch. Tradit Chin. Med..

[127] Chai H. (2016). Antitumor and Anti-Pulmonary Metastatic Mechanisms of Rhizoma Paridis Saponin. Master’s Thesis.

[128] Gao L., Li F., Kang L., Si Y., Wang H. (2009). Effects of the pariphyllin on expression of ICAM-1 and VCAM-1 in injury induced by hydrogen peroxide in the human umbiliar vein endothelial cell. China J. Mod. Med..

[129] Zouboulis C.C. (2004). Acne and sebaceous gland function. Clin. Dermatol..

[130] Huang X., Liu G., Guo J., Su Z. (2018). The PI3K/AKT pathway in obesity and type 2 diabetes. Int. J. Biol. Sci..

[131] Mu D., Li D., Li J., Yu H., Chen W., Liang J., Wang D., Li A., Qing Z., Zhang B. (2021). Long non-coding RNA HULC protects against atherosclerosis via inhibition of PI3K/AKT signaling pathway. Iubmb Life.

[132] Liu J. (2018). miR-338-3p Inhibits Skin Inflammation and Lung Cancer Progression by Regulating AKT Signaling Pathway. Ph.D. Thesis.

[133] Chen M., Gao S., Liu H., Tang Q., Luo H. (2019). Epigenetic modification regulation of Wnt pathway inhibitors to study the mechanism of polyphyllin V against colorectal cancer. Chin J. Col. Dis. (Electr. Ed.).

[134] Melnik B., Schmitz G. (2008). FGFR2 signaling and the pathogenesis of acne. JDDG J. Dtsch. Dermatol. Ges..

[135] Trenam C.W., Dabbagh A.J., Morris C.J., Blake D.R. (1991). Skin inflammation induced by reactive oxygen species (ROS): An in-vivo model. Br. J. Dermatol..

[136] Gaschler M.M., Stockwell B.R. (2017). Lipid peroxidation in cell death. Biochem. Biophys. Res. Commun..

[137] Adam W., Kurz A., Saha-Möller C.R. (2000). Peroxidase-catalyzed oxidative damage of DNA and 2′-deoxyguanosine by model compounds of lipid hydroperoxides: Involvement of peroxyl radicals. Chem. Res. Toxicol..

[138] Ayres S., Mihan R. (1978). Acne Vulgaris and Lipid Peroxidation: New Concepts in Pathogenesis and Treatment. Int. J. Dermatol..

[139] Li Y., Guan L., Chen L., Zhao M., Ding L., Meng C., Gao H., Wang Z. (2021). Qualitative and quantitative analysis of *Paris polyphylla* var. chinensis by UPLC-Q-TOF-MS/MS and HPLC. China J. Chin. Mater. Med..

[140] Gao Y., Yang L., Yang Y., Wang X. (2007). In vitro scavenging of reactive oxygen species and antioxidant effect of *Paris polyphylla* Sm extract. Chin. Tradit. Patent. Med..

[141] Jiang W., Li X., Wan J., Mei S., Fu X. (2021). HPLC Fingerprint of Total Saponins of Aerial Parts of *Paris polyphylla* var. yunnanenis. Mod. Chin. Med..

[142] Fu Y., Fu Q., Yang L., Bao Y., Tian Q., Gao Y., Liu X., Huang Q. (2023). Utilizing the above-ground extract of *Paris polyphylla* as a Nat antioxidant and antimicrobial additive in soap formulation. Biomass Convers. Biorefinery.

[143] Gao Z., Zhang D. (2019). A study on the protective effect of Paris polyphylla saponin on photoaging. Proceedings of the 13th Annual Academic Conference of the Chinese Society of Biotechnology and the 2019 National Biotechnology Conference, Chengdu, China, 2019.

[144] Xu L. (2013). Experimental and Clinical Studies on the Efficacy of a Complex Containing *Paris polyphylla* Extract and Hyaluronic Acid in Acne Vulgaris. Master’s Thesis.

[145] Fang T. (2023). Evaluation of Antimicrobial Activity of *Paris polyphylla* Extract against *Propionibacterium acnes* with Topical Dosage Form. Master’s Thesis.

[146] Wang Q., Zhou X., Zhao Y., Xiao J., Lu Y., Shi Q., Wang Y., Wang H., Liang Q. (2018). Polyphyllin I Ameliorates Collagen-Induced Arthritis by Suppressing the Inflammation Response in Macrophages through the NF-κB Pathway. Front. Immunol..

[147] Yang M., Zou J., Zhu H.M., Liu S.L., Wang H., Bai P., Xiao X. (2015). Paris saponin II inhibits human ovarian cancer cell-induced angiogenesis by modulating NF-κB signaling. Oncol. Rep..

[148] Pang D., Yang C., Li C., Zou Y., Feng B., Li L., Liu W., Luo Q., Chen Z., Huang C. (2020). Polyphyllin II inhibits liver cancer cell proliferation, migration and invasion through downregulated cofilin activity and the AKT/NF-κB pathway. Biol. Open.

[149] Qin X.J., Chen C.X., Ni W., Yan H., Liu H.Y. (2013). C-22-steroidal lactone glycosides from stems and leaves of *Paris polyphylla* var. yunnanensis. Fitoterapia.

[150] Tan L., Xiang M., Mi C., Li Z., Tian Z., Xiang B., Zhou W. (2017). Effect of Rhizoma Paridis Total Saponins on airway inflammation in a mouse model of asthma and the mechanism of the effect on airway inflammation in a mouse model of asthma. Chin. J. Geront..

[151] Li L., Liang C., Shan L., Xie X., Zhao D., Zhou M. (2009). Effects of Rhizoma Paridis Total Saponins on Levels of Cytokines in Blood Serum of Rats Subjected to Multiple Trauma. J. Liaoning Univ. Trad. Chin. Med..

[152] Lu R., Su X., Cai S. (2007). Optimization of Extraction Process of Saponins of *Paris polyphylla* with Orthogonal Design Method. Lishizhen Med. Mater. Med. Res..

[153] Sun Z., Zhang L., Li L., Tian J. (2007). Studies on the Extraction Process of Total Saponins from *Paris polyphylla* Smith. J. Chin. Med. Mater..

[154] Tan W., Dai J., Xiong Y., Yang M., Wang H. (2010). Extraction and Determination of Steroidal Saponins from *Paris polyphylla* Smith. J. Yunnan Univ. Nation (Nat. Sci. Ed.).

[155] Lu W., Yang G., Ye F., Hu Y. (2015). Optimization of Extraction Technology for Total Saponins in *Paris polyphylla* Smit by Orthogonal Design. China Pharm..

[156] Qian L., Chen Y. (2019). Study on Extraction and Stability of Active Components from *Paris polyphylla*. J. Huaihua Univ..

[157] Wang S., Su Y., Li H., Yang P., Wu P. (2019). Optimization of the reflux extraction process of steroidal saponins from paris herbs by response surface methodology. Shaanxi J. Agric. Sci..

[158] Liu B., Shao M., Yang Y., Yang G. (2020). Optimal design of an orthogonal test for the extraction of four saponins from Paris. Lis. Med. Mater. Med. Res..

[159] Luo Y., Liu S., Huang S. (2010). Ultrasonic extraction of total saponins from *Paris polyphylla* smith by orthogonal design. Pract. Pharm. Clin. Remedies.

[160] Liu J., Duan Z., Duan B., Xia C. (2016). Ultrasonic Extraction Technology of Four Kinds of Polyphyllin in *Paris vietnamensis*. J. Anhui Agric. Sci..

[161] Li W., Peng Y., Li Y., Chen X. (2010). Ultrasonic Extraction of *Paris polyphylla* saponins from paris. Chin. Tradit. Patent. Med..

[162] Wang F., Ma X., Li Z., Yuan Y., Qu L. (2017). Ultrasonic Extraction of Total Saponins and Content Difference of Active Components from Colloidal and Amyloid *Paris polyphylla*. Pharm. J. Chin People’s Lib. Army.

[163] Lan J., Yang M., Ma J. (2006). Study of the extraction process in Paris. Chin. Tradit. Herb. Drugs.

[164] Yu Z., Liu Y., Li H., Li F. (2013). Study on the extraction of total saponins from *Paris polyphylla* smith by Microwave. Lis. Med. Mater. Med. Res..

[165] Xiao X.H., Yuan Z.Q., Li G.K. (2014). Separation and purification of steroidal saponins from *Paris polyphylla* by microwave- assisted extraction coupled with countercurrent chromatography using evaporative light scattering detection. J. Sep. Sci..

[166] Zhang S.Q., Zhang J.S., Wang C.Z. (2007). Extraction of steroid saponins from *Paris polyphylla* Sm. var. *yunnanensis* using novel ultrahigh pressure extraction technology. Pharm. Chem. J..

[167] Tu Y., Jiang L., Yang Y. (2018). Study on the Process for Supercritical Carbon Dioxide Extraction of Pennogenin Ingredients of *Parispolyphylla* Sm. var. *yunnanensis*. J. Kun Univ. Sci. Tech. (Nat. Sci. Ed.).

[168] Tong L., Cai H. (2012). Enzymatic Extraction Technology of Total Saponins in Paris. Aca. Peri. Farm. Pro. Pro..

[169] Nian S., Zhang H., Zheng Y. (2006). Studies on Water-extracting Technology from Rhizoma Paris. J. Yunnan Univ. Tradit. Chin. Med..

[170] Negi J.S., Bisht V.K., Bhandari A.K., Bhatt V.P., Singh P., Singh N. (2014). Paris polyphylla: Chemical and biological prospectives. Anti-Cancer Agents Med. Chem..

[171] Liu N., Chen L., Zhang Y., Zhang L., Fan J. (2019). Response surface optimization on ultrasound-assisted extraction of hemp cannabinoids and study on anti-oxidation property. Mod. Chem. Ind..

[172] Sadowska-Rociek A., Surma M., Cieslik E. (2014). Comparison of different modifications on QuEChERS sample preparation method for PAHs determination in black, green, red and white tea. Environ. Sci. Pollut. R..

[173] Cheok C.Y., Salman H.A.K., Sulaiman R. (2014). Extraction and quantification of saponins: A review. Food Res. Int..

[174] Jun X. (2013). High-Pressure Processing as Emergent Technology for the Extraction of Bioactive Ingredients from Plant Materials. Crit. Rev. Food Sci..

[175] Seo Y.C., Choi W.Y., Kim J.S., Yoon C.S., Lim H.W., Cho J.S., Ahn J.H., Lee H.Y. (2011). Effect of ultra high pressure processing on immuno-modulatory activities of the fruits of Rubus coreanus Miquel. Innov. Food Sci. Emerg..

[176] Ning N., Zhou J. (2008). Research progress of ultra-high pressure extraction technology in Chinese medicine extraction. Tianjin Pharm..

[177] Pan S.Y., Chiang P.C., Pan W.B., Kim H. (2018). Advances in state-of-art valorization technologies for captured CO_2_ toward sustainable carbon cycle. Crit. Rev. Environ. Sci. Technol..

[178] Bhattacharjee P., Chatterjee D., Singhal R.S. (2012). Supercritical Carbon Dioxide Extraction of Squalene from *Amaranthus paniculatus*: Experiments and Process Characterization. Food Bioprocess Technol..

[179] Ye F., Yang G., Li Z., Wang G. (2011). Overview of the extraction process and quality control studies of *Paris polyphylla* saponins. China Pharm..

[180] Gao Y.H., Liu C., Yao F., Chen F.S. (2021). Aqueous enzymatic extraction of peanut oil body and protein and evaluation of its physicochemical and functional properties. Int. J. Food Eng..

[181] Jian Q., Gao J., Chen Z., Jing M., Xue J., Liu X. (2019). Extraction of active ingredient from Chinese materia medica by enzyme and enzyme coupling technique. J. Gansu Univ. Chin. Med..

[182] Wang Z., Yang L., Zeng X., Li P., Zhang X. (2013). Application Progress on Enzymatic Extraction Technology in Extraction of Chemical Compositions of Chinese Medicine. World Chin. Med..

