# Supplementary material for: Saponins of Paris polyphylla for the Improvement of Acne: Anti-Inflammatory, Antibacterial, Antioxidant and Immunomodulatory Effects
Source: Molecules. 2024 Apr 15;29(8):1793. doi: 10.3390/molecules29081793 (PMC11052371; doi:10.3390/molecules29081793)
Supplement: Supplementary file 1 [file molecules-29-01793-s001.zip › molecules-2939481-supplementary.pdf]

Supplementary Materials

Saponins of *P. polyphylla* for the Improvement of Acne: Anti-Inflammatory, Antibacterial, Antioxidant and Immunomodulatory Effects

Luyao Li [1,2,3], Jiachan Zhang [1,2,3,*], Wenjing Cheng [1,2,3], Feiqian Di [1,2,3], Changtao Wang [1,2,3] and Quan An [4]

[1] College of Light Industry Science and Engineering, Beijing Technology & Business University, Beijing 100048, China
[2] Beijing Key Lab of Plant Resource Research and Development, Beijing 100048, China
[3] Institute of Cosmetic Regulatory Science, Beijing 100048, China
[4] Yunnan Baiyao Group Co., Ltd. Yunnan 650000, China
* Correspondence: xiaochan8787@163.com; Tel.: +86-13426258535

S1. Organic Solvent Extraction Methods

Organic solvent extraction is a traditional process widely used in many medicinal plant extractions, apart from *P. polyphylla* saponins. The typical organic solvent extraction method is RE. Because of its high extraction efficiency, safety, and low cost, ethanol is typically used as the solvent. However, with a relatively high temperature, RE usually takes a long extraction time. In order to improve the extraction rate and shorten the extraction time, USA-SE, UHPA-SE, and MWA-SE are developed.

However, the sugar group of *P. polyphylla* saponins uses L-rhamnose, and its polarity is weak [1]. Therefore, a long period of high-temperature extraction makes it easy to break the sugar chain, leading to *P. polyphylla* saponin degradation. Moreover, the accelerated solvent volatilization changes the polarity of the solid–liquid system, thus affecting the solubility of *P. polyphylla* saponins. Therefore, it is necessary to explore the proper extraction parameters, such as solvent selection, solvent concentration, extraction temperature, extraction time, and the number of extractions.

Here, we described the characteristics of some traditional and newly developed extraction methods.

S1.1. Reflux extraction (RE)

RE is the most conventional method for extracting *P. polyphylla* saponins, with the advantages of low cost, high yield, and ease of industrialization [2]. However, obtaining a high extraction rate usually takes a long time.

The traditional RE parameters are types of solvents, solvent dosages, temperatures, and extraction times. Changes in extraction conditions significantly affect the extraction of *P. polyphylla* saponins. Literature research revealed that ethanol (70%-90%) was chosen as the solvent for the extraction and the extraction rate was relatively high (12.74%) when extracted at

90°C [3,4,5], with two extractions being optimal [5-9] compared to the other temperatures. Meanwhile, pretreatment based on sample pulverization fineness [10] also significantly affects the yield and quality of *P. polyphylla* saponins.

S1.2. Ultrasonic-assisted solvent extraction (USA-SE)

USA-SE uses ultrasonic technology as an assisted technique to destroy cell walls, accelerate solvent penetration into the cells, and promote the dissolution of target compounds into the medium; as such, it is considered a promising and green technology [11,12,13]. The solvents usually used to extract *P. polyphylla* saponins include water, n-butanol, ethanol, and methanol. Recent literature studies have reported that the maximum extraction rate of *P. polyphylla* saponins reached 9.50% [9]. USA-SE showed a significantly shorter extraction time compared to other extraction methods. Lan et al. [14] compared the extraction yield of *P. polyphylla* saponins using three techniques (immersion, RE, and USA-SE) and found no significant difference between USA-SE and RE methods, while the extraction time of USA-SE (30 min) was significantly lower than that of RE (240 min) and the immersion (24 h). Similar results were reported by Man et al. [15]

Therefore, USA-SE can be preferred for the extraction of *P. polyphylla* saponins when considering the extraction rate and extraction time of *P. polyphylla* saponins.

S1.3. Microwave-Assisted Solvent Extraction (MWA-SE)

MWA-SE has low reagent consumption, low pollution, and high extraction efficiency, and it is energy- and time-saving [16]. Unlike RE and USA-SE, MWA-SE directly applies energy to the target material, ensuring rapid energy transfer and full utilization, reducing pollution and work intensity.

Several factors are involved in MWA-SE, such as liquid–solid ratio, microwave power, and treatment time. Generally, microwave energy is higher than ultrasonic waves when dealing with the same target samples. This might be the reason for a relatively higher yield and a shorter treatment period of MWA-SE than USA-SE. Under the proper MWA-SE conditions with a wave intensity at 300-500W [17], the extraction yield of *P. polyphylla* saponins was higher than that of USA-SE (100-200W) [9,14,18]. Moreover, the extraction time of MWA-SE (5-25 min) was shorter than that of USA-SE (20-60 min). Yu et al. [19] compared RE and MWA-SE through a single-factor test on total *P. polyphylla* saponin extraction. It was found that the extraction rate using MWA-SE (11.6%) was higher than that using RE (11.3%), and the extraction time (20 min) was much lower than that of RE (90 min).

All the results above confirmed that MWA-SE obtained a higher extraction rate and a significantly shorter extraction time. Therefore, MWA-SE can preferentially extract *P. polyphylla* saponins as a more efficient, environmentally friendly, and time-saving method.

S2. Physical Extraction Methods

S2.1. Ultrahigh-Pressure-Assisted Solvent Extraction (UHPA-SE)

UHPA-SE, also known as ultrahigh-pressure cold pressing, is an effective technology for extracting natural bioactive substances. Ultrahigh pressure (UHP) ranges from 100 MPa to 600 MPa. The typical ultrahigh pressure exhibits the advantages of a high extraction rate, short processing time, low energy consumption, and high safety [20]. In addition, UHPA-SE over-regulates pressure and causes cell membrane disruption due to the difference in internal and external osmotic pressure, thus improving the extraction rate of *P. polyphylla* saponins [21]. A study compared the extraction methods of several traditional extracts of *P. polyphylla* saponin monomers using the same extraction solvent (90% ethanol) [22]. UHPA-SE caused the worst damage to plant cells, and its extraction rate (1.164%) was the highest among the discussed methods, such as MWA-SE, USA-SE, and RE (1.139%, 1.1135%, and 0.988%, respectively). In addition, the extraction time (2 min) of UHPA-SE was the least among those conventional extraction methods (15min to 5 days) [22].

High pressure is thought to have caused the starch to melt, preventing the saponin from dissolving into the extraction solvent [23]. Generally, 400 MPa of pressure tends to be an optimal parameter. However, when the pressure reaches 500 MPa, the extraction rate of *P. polyphylla* saponins from rhizomes decreases [22].

UHPA-SE can be used as a high-efficiency extraction technology for the active components of medicinal plants, paving the way for modernizing traditional Chinese medicine pharmaceutical technology.

S2.2. Supercritical Fluid CO_2 Extraction (SFE-CO_2)

SFE-CO_2 is an efficient, high-yield, environmentally friendly, and comprehensive separation technology. SFE-CO_2 has the advantages of strong fat solubility, good mass transfer performance, non-toxicity, and high relative molecular weight [24].

Like UHPA-SE, pressure is also a key factor in controlling the fluid state. As the pressure increases, the density of the fluid increases, leading to a decrease in the intermolecular mass transfer distance and an increase in the mass transfer efficiency of solutes and solvents. However, a higher pressure weakens the interaction between solutes and solvents, reducing the extraction rate and being detrimental to extract extraction [25]. Tu et al. [26] conducted an experimental study on the pressure of 100-600bar and found that the pressure of 350 bar was the best extraction condition, with a 15.96% extraction rate of *P. polyphylla* saponins.

Since organic solvent extraction (RE, MWA-SE, and USA-SE) and UHPA-SE usually require organic solvents as solvents and may be accompanied by heating methods, which can easily cause organic residues of the drug and affect the chemical stability of the active ingredients [21], thus affecting their

pharmacological effects. Therefore, SFE-CO_2 can be an effective method for extracting high-purity *P. polyphylla*.

S3. Aqueous Enzymatic Extraction (AEE)

Traditional extraction processes usually use organic solvents which are not conducive to purifying P. polyphylla saponins. Furthermore, the procedures can cause some harm to health and the environment.

Biological methods have been developed. Enzymes can use hydrolysate-related substrates for small molecules. The purpose of AEE is to hydrolyze the cellulose, proteins, or other fibers with the help of enzymes to break the network structure, thus obtaining a high extraction rate of target compounds. *P. polyphylla* contains fibers, starch, colloids, and proteins [27]. Selecting specific enzymes to break down starch, mucilage, and protein, which aid in filtering and purifying the target product, increasing extraction rates.

There are lots of enzymes used in the extraction of saponins, such as proteases [28], cellulose, hemicellulose, and pectinase [29]. The cell wall of plants is mainly composed of cellulose; the addition of cellulase during extraction can promote the hydrolysis of cellulose β-D-glucosidic bonds, destroy the plant cell wall, and maximize the leaching of active ingredients from the cells [29,30]. Thus, cellulase is most suitable for the extraction of medicinal ingredients.

Enzymes can also cooperate with other standard techniques to increase the target yield. Tong et al. [31] used cellulase treatment followed by ultrasound extraction to overcome the problem of the low yield of total saponins from *P. polyphylla*. The extraction was carried out via ultrasonic extraction, and the extraction rate of total saponins from *P. polyphylla* reached 1.66%.

Moreover, the use of enzymes, especially combining different enzymes, could improve the filtration problem during the separation process. For example, Nian et al. [32] found that adding enzyme A and enzyme B alternately in the aqueous extraction process could solve the difficult filtration problem and increase the extraction rate from 67.78% to 84.24%.

We believe that the use of enzymes and combination techniques will benefit the production of *P. polyphylla* saponins with the development of the AEE study.

References

1. Negi, J.S.; Bisht, V.K.; Bhandari, A.K.; Bhatt, V.P.; Singh, P.; Singh, N. Paris polyphylla: chemical and biological prospectives. *Anti-Cancer Agent. Me.* **2014**, *14*, 833.
2. Lu R.; Su X.; Cai S. Optimization of Extraction Process of Saponins of Paris Polyphylla with Orthogonal Design Method. *Lishizhen. Med. Materia. Medica Res.* **2007**, 1044-1045.
3. Qian L.; Chen Y. Study on Extraction and Stability of Active Components from Paris Polyphylla. *J. Huaihua Uni.* **2019**, *38*, 11-16.
4. Tan W.; Dai J.; Xiong Y.; Yang M.; Wang H. Extraction and Determination of Steroidal Saponins from Paris Polyphylla Smith. *J. Yunnan. Uni. National. (Natur. Sci. Edit)* **2010**, *19*, 127-129.
5. Liu B.; Shao M.; Yang Y.; Yang G. Optimal design of an orthogonal test for the extraction of four saponins from Paris. *Lishizhen. Med. Materia. Medica Res.* **2020**, *31*, 1591-1594.
6. Sun Z.; Zhang L.; Li L.; Tian J. Studies on the Extraction Process of Total Saponins from Paris polyphylla Smith. *J. Chin. Med. Mater.* **2007**, 726-729.

7. Lu W.; Yang G.; Ye F.; Hu Y. Optimization of Extraction Technology for Total Saponins in Paris Polyphylla Smit by Orthogonal Design. *China. Pharm.* **2015**, *18*, 1484-1487.
8. Wang S.; Su Y.; Li H.; Yang P.; Wu P. Optimization of the reflux extraction process of steroidal saponins from paris herbs by response surface methodology. *Agric. Sci.* **2019**, *65*, 46-52.
9. Luo Y.; Liu S.; Huang S. Ultrasonic extraction of total saponins from paris polyphylla smith by orthogonal design. *Practical Pharm. Clin. Remedies.* **2010**, *13*, 185-187.
10. Jiang W.; Li X.; Wan J.; Mei S.; Fu X. HPLC Fingerprint of Total Saponins of Aerial Parts of Paris polyphylla var.yunnanenis. *Mod. Chin. Med* .**2021**, *23*, 57-61.
11. Liu N.; Chen L.; Zhang Y.; Zhang L.; Fan J. Response surface optimization on ultrasound-assisted extraction of hemp cannabinoids and study on anti-oxidation property. *Mod. Chem. Ind.* **2019**, *39*, 144-149.
12. Li W.; Peng Y.; Li Y.; Chen X. Ultrasonic Extraction of Paris polyphylla saponins from paris. *Chin. Tradit. Patent. Med.* **2010**, *32*, 218-220.
13. Liu J.; Duan Z.; Duan B.; Xia C. Ultrasonic Extraction Technology of Four Kinds of Polyphyllin in Paris vietnamensis. *J. Anhui Agr. Sci.* **2016**, *44*, 158-160.
14. Lan J.; Yang M.; Ma J. Study of the extraction process in Paris. *Chin. Tradit. Herb. Drugs*. **2006**, 871-872.
15. Wang F.; Ma X.; Li Z.; Yuan Y.; Qu L. Ultrasonic Extraction of Total Saponins and Content Difference of Active Components from Colloidal and Amyloid Paris Polyphylla. *Pharmaceutical. J. Chin. People's. Liberat. Army*. **2017**, *33*, 315-318.
16. Sadowska-Rociek, A.; Surma, M.; Cieslik, E. Comparison of different modifications on QuEChERS sample preparation method for PAHs determination in black, green, red and white tea. *Environ. Sci. Pollut. R.* **2014**, *21*, 1326-1338.
17. Xiao, X.H.; Yuan, Z.Q.; Li, G.K. Separation and purification of steroidal saponins from Paris polyphylla by microwave- assisted extraction coupled with countercurrent chromatography using evaporative light scattering detection. *J. Sep. Sci.* **2014**, *37*, 635-641.
18. Cheok, C.Y.; Salman, H.A.K.; Sulaiman, R. Extraction and quantification of saponins: A review. *Food Res. Int.* **2014**, *59*, 16-40.
19. Yu Z.; Liu Y.; Li H.; Li F. Study on the extraction of total saponins from paris polyphylla smith by Microwave. *Lishizhen. Med. Materia. Medica Res.* **2013**, *24*, 833-834.
20. Jun, X. High-Pressure Processing as Emergent Technology for the Extraction of Bioactive Ingredients From Plant Materials. *Crit. Rev. Food Sci.* **2013**, *53*, 837-852.
21. Seo, Y.C.; Choi, W.Y.; Kim, J.S.; Yoon, C.S.; Lim, H.W.; Cho, J.S.; Ahn, J.H.; Lee, H.Y. Effect of ultra high pressure processing on immuno-modulatory activities of the fruits of Rubus coreanus Miquel. *Innov. Food Sci. Emerg.* **2011**, *12*, 207-215.
22. Zhang, S.Q.; Zhang, J.S.; Wang, C.Z. Extraction of steroid saponins from Paris polyphylla Sm. var. yunnanensis using novel ultrahigh pressure extraction technology. *Pharm. Chem. J.* **2007**, *41*, 424-429.
23. Ning N.; Zhou J. Research progress of ultra-high pressure extraction technology in Chinese medicine extraction. *Tianjin. Pharm.* **2008**, 62-64.
24. Pan, S.Y.; Chiang, P.C.; Pan, W.B.; Kim, H. Advances in state-of-art valorization technologies for captured CO_2 toward sustainable carbon cycle. *Crit. Rev. Env. Sci. Tec.* **2018**, *48*, 471-534.
25. Bhattacharjee, P.; Chatterjee, D.; Singhal, R.S. Supercritical Carbon Dioxide Extraction of Squalene from Amaranthus paniculatus: Experiments and Process Characterization. *Food. Bio. Tech.* **2012**, *5*, 2506-2521.
26. Tu Y.; Jiang L.; Yang Y. Study on the Process for Supercritical Carbon Dioxide Extraction of Pennogenin Ingredients of Parispolyphylla SM. var. yunnanensis. *J. Kunming. Uni. Sci. Tech. (Natur. Sci. Edit).* 2018, 43, 98-104.
27. Ye, F.; Yang, G.; Li, Z.; Wang, G. Overview of the extraction process and quality control studies of Paris polyphylla saponins. *China. Pharm.***2011**, 14, 1207-1209.
28. Gao, Y.H.; Liu, C.; Yao, F.; Chen, F.S. Aqueous enzymatic extraction of peanut oil body and protein and evaluation of its physicochemical and functional properties. *Int. J. Food Eng.* **2021**, *17*, 897-908.
29. Jian Q.; Gao J.; Chen Z.; Jing M.; Xue J.; Liu X. Extraction of active ingredient from Chinese materia medica by enzyme and enzyme coupling technique. *J. Gansu. Uni. Chin. Med.* **2019**, *36*, 79-82.
30. Wang Z.; Yang L.; Zeng X.; Li P.; Zhang X. Application Progress on Enzymatic Extraction Technology in Extraction of Chemical Compositions of Chinese Medicine. *World. Chin. Med.* **2013**, *8*, 104-106.
31. Tong L.; Cai H. Enzymatic Extraction Technology of Total Saponins in Paris. *Acad. Periodi, Farm. Prod. Proc.* **2012**, 62-67.
32. Nian S.; Zhang H.; Zheng Y. Studies on Water-extracting Technology From Rhizoma Paris. *J. Yunnan. Uni. Tradit. Chin. Med.* **2006**, 13-16.
